# The role of electronic excited states in the spin-lattice relaxation of spin-1/2 molecules

**DOI:** 10.1126/sciadv.adr0168

**Published:** 2025-02-12

**Authors:** Lorenzo A. Mariano, Vu Ha Anh Nguyen, Jonatan B. Petersen, Magnus Björnsson, Jesper Bendix, Gareth R. Eaton, Sandra S. Eaton, Alessandro Lunghi

**Affiliations:** ^1^ School of Physics, AMBER and CRANN Institute, Trinity College, Dublin 2, Ireland.; ^2^ Department of Chemistry, The University of Manchester, Manchester M13 9PL, UK.; ^3^ Department of Chemistry, University of Copenhagen, DK-2100 Copenhagen, Denmark.; ^4^ Department of Chemistry and Biochemistry, University of Denver, Denver, CO 80210, USA.

## Abstract

Magnetic resonance is a prime method for the study of chemical and biological structures and their dynamical processes. The interpretation of many of these experiments relies on understanding how the spin of unpaired electrons exchanges energy with their environment, or lattice, and relaxes to equilibrium. Here, we overcome the common use of effective spin Hamiltonians to describe spin-lattice relaxation in spin-1/2 and apply ab initio open quantum systems theory to their full molecular electronic wavefunction. Simulations for two Cr(V) coordination compounds under this framework show a marked improvement in accuracy and demonstrate that relaxation in spin-1/2 molecules is enabled by virtual transitions to molecular excited states with energy approaching 20,000 cm^–1^. This work establishes a connection between the original theory of Van Vleck and modern electronic structure methods, ultimately exemplifying the urgency of further advancing an ab initio approach to spin-lattice relaxation.

## Teaser

Ab initio calculations explain spin-lattice relaxation is spin-1/2 molecules.

## INTRODUCTION

The ubiquitous presence of spin in chemical compounds, because of the presence of atoms with nuclear spins or unpaired electrons, makes magnetic resonance a central experimental technique for the study of chemical and biological processes. In particular, the time it takes for a spin to reach its thermal equilibrium state, namely, the spin relaxation time, provides unique insights into the interactions between the spin and its chemical environment. For instance, the study of spin relaxation time can be exploited to determine the aggregation state of bio-molecules (*1*), their folding dynamics (*2*), the presence of analytes in solutions (*3*), and enhanced in vivo imaging techniques (*4*). In recent years, relaxometry experiments have also become a central tool of quantum sensing (*5*), where the relaxation of highly coherent spin states is used to probe magnetic fields and magnetic states with unprecedented sensitivity (*6*). The establishment of a quantitative theoretical framework of spin relaxation is essential to correctly interpret and design these experiments, but it is yet to be fully achieved. In particular, fundamental questions about relaxation at temperatures above ~20 K remain open for what appears as the most fundamental unit: a spin-1/2.

Early attempts to establish a quantum theory of electron spin relaxation in solid state are summarized in books by Abragam and Bleaney (*7*), Poole and Farach (*8*), and Standley and Vaughn (*9*). That theory is based on the seminal work of Van Vleck (*10*), who proposed a perturbative treatment of how the thermal motion of atoms inside a crystalline lattice, i.e., the phonons, affects the crystal field of unpaired electrons located in the 3*d* or 4*f* shell of an ion. Such interaction was initially referred to as orbit-lattice interaction and was established as the main driver for spin relaxation above liquid He temperature. With modern electronic structure theory at least 25 years in the future, the approach of Van Vleck was based on strong assumptions about the way lattice vibrations interact with the ion’s crystal field. For instance, a Debye distribution of phonon energies with a cutoff at the Debye temperature (θ_D_) was assumed, and the orbit-lattice interaction was estimated as purely electrostatic in origin. Although this early attempt set the stage for any subsequent theoretical treatment of spin relaxation, these approximations were too drastic to permit even semiquantitative predictions of experimental observations. So much so that 20 years later Orbach proposed to leave a first-principles approach behind in favor of a phenomenological one (*11*). This early literature eventually led to the modeling of the temperature, *T*, dependence of relaxation time, *T*_1_, in terms of phenomenological expressions describing what is known as the direct, Raman, local mode, and Orbach processes. Important lessons on the relaxation of spin-1/2 molecules have been learned by applying this phenomenological approach to experiments, e.g., (i) spin-lattice relaxation rates increase as the deviation of the effective *g*-factor from the free electron value of 2.0023 increases (*12*, *13*). Since spin-orbit coupling determines such shifts, changes in *g*-factor values are useful proxies for the effect of spin-orbit coupling in spin relaxation (*14*). (ii) *T*_1_ is the same at X-band and Q-band or W-band and *T* > 20 K for Cu(II) and vanadyl complexes (*15*, *16*). (iii) *T*_1_ is the same for the *I* = 0 and *I* = 3/2 isotopes of Cr(V) coordination compounds (*17*).

Despite its utility, such a phenomenological theory of spin-lattice relaxation and analysis of data in terms of adjustable parameters can only partly assist the interpretation of relaxometry experiments due to the fact that it is not a predictive tool and does not make it possible to unequivocally determine the leading relaxation mechanism, nor the interactions at the origin of relaxation itself. Faced with this discouraging situation, one must of necessity reintroduce a first-principles approach. This ab initio theory of spin relaxation has been recently proposed (*18*) and has already provided a successful prediction of both one- and two-phonon spin relaxation rates for a diverse range of systems, ranging from coordination compounds of 3*d* and 4*f* ions with long relaxation times (*19*–*22*), known as single-molecule magnets, to solid-state quantum sensors based on color centers (*23*, *24*), such as defects in diamond and hexagonal boron nitride. However, a common denominator for all these compounds is a spin moment larger than 1/2 and energy levels defined by zero-field splitting.

A key characteristic of this ab initio approach is that it is based on effective Hamiltonian theories (*25*), where only the first 2*J* + 1 electronic states are assumed to play a role in relaxation, with *J* being the total angular momentum of the ion’s unpaired electrons. In this framework, the original orbit-lattice interaction is recast in terms of lattice modulations of such an effective Hamiltonian, which for spin-1/2 corresponds to a spin Hamiltonian including Zeeman and hyperfine interactions (*26*, *27*). Under this assumption, ab initio methods have failed to explain all of the experimental observations for spin-1/2 listed above, with the lattice modulation of the Zeeman interaction failing to explain the timescale of relaxation and the absence of a correlation with the external magnetic field intensity (*19*, *27*), and the modulation of the hyperfine interaction failing to reproduce the angular dependence of relaxation rates (*16*) and the correlation with spin-orbit coupling strength (*28*).

Despite decades of theoretical and computational efforts, we are therefore left with two seemingly incompatible formalisms. On the one hand, the original proposal of Van Vleck is generally credited as the correct explanation of the relaxation mechanism of spin-1/2, but it has never been successfully used to make quantitative predictions. On the other hand, ab initio theories, supposedly quantitative by nature, have failed to reproduce all the experimental evidence. Overall, such a state of affairs casts serious doubts on our understanding of this fundamental physical process and must be urgently addressed.

Here, we show that this conundrum can be solved by going beyond the use of effective spin Hamiltonians and by using the full electronic Hamiltonian, as recently advocated (*16*). This allows explicit inclusion of high-energy electronic excited states capable of contributing as virtual states to spin relaxation. We show that this computational framework largely improves the accuracy of predictions of relaxation times for spin-1/2 coordination compounds as measured by electron paramagnetic resonance (EPR) inversion recovery experiments, advancing the establishment of a complete ab initio theory of spin relaxation and improving our understanding of this fundamental physical process.

The article is structured as follows: (i) the theory of spin-lattice relaxation and its use in interpreting experiments is reviewed, (ii) synthesis and EPR characterization of two prototypical spin-1/2 Cr(V) coordination compounds are presented, and (iii) ab initio simulations are applied to the same compounds to determine the correct relaxation mechanism for spin-1/2 molecules.

## RESULTS

### Spin-lattice relaxation theory

In this section, we aim to introduce the main theoretical concepts behind spin-lattice relaxation and how these have been used for interpreting experimental results and more recently as a basis for ab initio simulations. We do not attempt to provide a full account of such a theory, and the interested reader is directed toward classical books (*7*, *9*) on the topics as well as recent contributions (*18*).

### Spin-lattice interactions

We start by defining the total Hamiltonian describing the electronic open system interacting with the motion of the atoms in the lattice

$${\hat{H}}_{\text{tot}}={\hat{H}}_{e}+{\hat{H}}_{\text{ph}}+{\hat{H}}_{e-\text{ph}}$$ (1)

where the electronic Hamiltonian ${\hat{H}}_{e}={\hat{H}}_{\text{BO}}+{\hat{H}}_{\text{soc}}$ includes the effects of both the nonrelativistic Born-Oppenheimer Hamiltonian, ${\hat{H}}_{\text{BO}}$, and the spin-orbit coupling Hamiltonian, ${\hat{H}}_{\text{soc}}$. The latter operators describe the electrons at the equilibrium nuclear coordinates. The second term of the right-hand side of Eq. 1 refers to the Hamiltonian of the nuclei under the Born-Oppenheimer approximation. Under the additional assumption that the effective potential felt by the nuclei is harmonic, ${\hat{H}}_{\text{ph}}$ then coincides with a set of independent quantum harmonic oscillators, namely, phonons, and takes the form

$${\hat{H}}_{\text{ph}}=\sum_{\alpha }\hbar \omega_{\alpha }\left({\hat{n}}_{\alpha }+\frac{1}{2}\right)$$ (2)

where ℏω_α_ is the energy quanta of the phonon *Q*_α_, and ${\hat{n}}_{\alpha }$ is the operator describing the number of such phonons. Without any loss of generality, the index α in Eq. 2 could refer to both band and reciprocal space indexes of phonons. Finally, the last term in Eq. 1 is the coupling between electrons and phonons, which accounts for the modulation of the electronic interactions during the lattice dynamics. Mathematically, this takes the form of a Taylor expansion of ${\hat{H}}_{e}$ with respect to the atomic displacements associated with the phonons *Q*_α_

$${\hat{H}}_{e-\text{ph}}=\sum_{\alpha }\left(\frac{\partial {\hat{H}}_{e}}{\partial Q_{\alpha }}\right)Q_{\alpha }+\sum_{\alpha \leq \beta }\left(\frac{\partial^{2}{\hat{H}}_{e}}{\partial Q_{\alpha }\partial Q_{\beta }}\right)Q_{\alpha }Q_{\beta }$$ (3)

The coefficients appearing in Eq. 3 often take the name of vibronic coupling coefficients or electron-phonon coupling coefficients.

Over the years, the possibility of reformulating this problem in terms of effective Hamiltonians has been advanced (*29*–*32*). In such an approach, the electronic Hamiltonian of a spin-1/2 molecule is substituted with the effective spin Hamiltonian of the form

$${\hat{H}}_{s}=\mu_{B}\;\vec{S}\cdot g\cdot \vec{B}+\vec{S}\cdot A\cdot \vec{I}$$ (4)

where the electron spin operator $\vec{S}$ is coupled to the external magnetic field $\vec{B}$ and the ion’s nuclear spin $\vec{I}$ through the Bohr’s magneton, μ_B_, the Lande tensor, **g**, and the hyperfine coupling tensor, **A**, respectively. Here, we exclude dipolar interactions with any other spin. The latter are known to contribute to relaxation only in the limits of low temperature or high concentration of magnetic ions in the lattice. Neither condition applies to our study. Accordingly, the same transformation is also applied to the Hamiltonian describing the coupling between the unpaired electrons and the lattice, ${\hat{H}}_{e-\text{ph}}\to {\hat{H}}_{s-\text{ph}}$, where

$${\hat{H}}_{s-\text{ph}}=\sum_{\alpha }\left(\frac{\partial {\hat{H}}_{s}}{\partial Q_{\alpha }}\right)Q_{\alpha }+\sum_{\alpha \leq \beta }\left(\frac{\partial^{2}{\hat{H}}_{s}}{\partial Q_{\alpha }\partial Q_{\beta }}\right)Q_{\alpha }Q_{\beta }$$ (5)

${\hat{H}}_{s-\text{ph}}$ takes the name of spin-lattice or spin-phonon interaction to emphasize that the electron’s orbital degrees of freedom are not an explicit part of the model anymore. Although intuitive, the justification of a spin-phonon Hamiltonian in the form of Eq. 5 is far from trivial and it has been noted that additional terms might be necessary to have an exact mapping with the full quantum mechanical treatment of Eq. 3 (*33*, *34*).

### Spin-lattice dynamics

Open quantum systems theory provides a framework for studying the time evolution of a system with a Hamiltonian of the form

$$\hat{H}={\hat{H}}_{0}+{\hat{H}}_{\text{ph}}+\sum_{\alpha }{\hat{V}}^{\alpha }Q_{\alpha }+\sum_{\alpha \leq \beta }{\hat{V}}^{\alpha \beta }Q_{\alpha }Q_{\beta }$$ (6)

where ${\hat{H}}_{0}$ corresponds to either the real electronic Hamiltonian or the effective spin Hamiltonian, depending on the framework used, and ${\hat{V}}^{\alpha }$ and ${\hat{V}}^{\alpha \beta }$ are a shorthand notation for the derivatives of ${\hat{H}}_{0}$ as they appear in Eqs. 3 and 5. Previous works showed that a perturbative approach to this problem leads to a series of contributions to *T*_1_ due to one- and two-phonon processes (*18*, *19*). It is well established that two-phonon processes are responsible for relaxation at high temperatures for spin-1/2 (*7*), and thus, we only focus on these.

There are two different contributions to two-phonon relaxation

$$\frac{1}{2T_{1}}=\Gamma_{I}+\Gamma_{\text{II}}$$ (7)

**Fig. 1.**
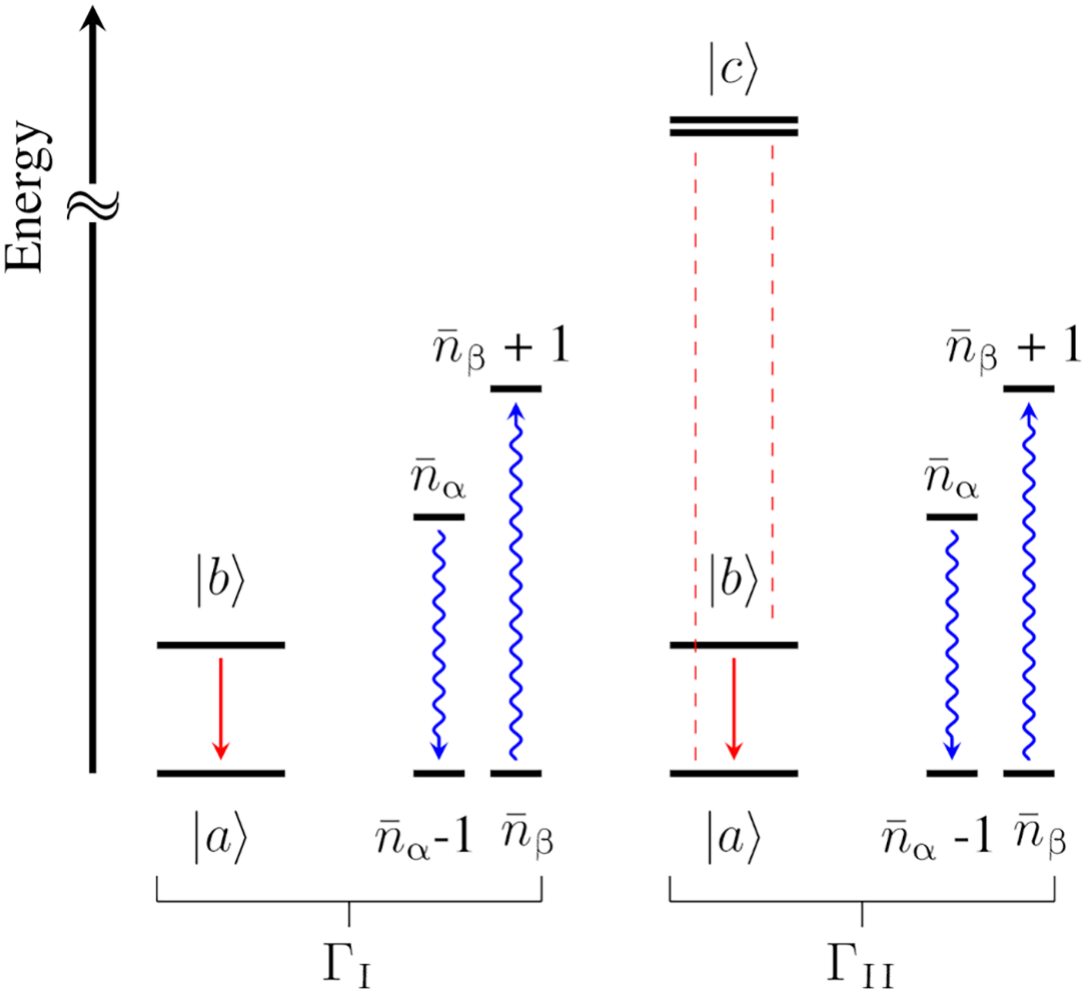
Schematic representation of spin-phonon transitions. The left panel describes a spin transition due to a two-phonon process, where one phonon is emitted and one is simultaneously absorbed. The right panel describes a spin transition among the same two levels, where now the two-phonon process occurs because of virtual transitions (red dashed lines) to the excited electronic states ∣*c*〉. The latter are well separated in energy from both spin and phonon excitations.

Γ_I_ arises from combining quadratic terms in *Q*_α_ of Eq. 6 with second-order perturbation theory, and reads

$$\Gamma_{I}=\frac{\pi }{2\hbar^{2}}\sum_{\alpha \geq \beta }{|V_{\text{ab}}^{\alpha \beta }|}^{2}\;G\left(\omega_{\text{ab}},\omega_{\alpha },\omega_{\beta }\right)$$ (8)

where

$$G\left(\omega_{\text{ba}},\omega_{\alpha },\omega_{\beta }\right)=\delta \left(\omega_{\text{ba}}-\omega_{\alpha }+\omega_{\beta }\right){\bar{n}}_{\alpha }\left({\bar{n}}_{\beta }+1\right)$$ (9)

and $V_{\text{ab}}^{\alpha \beta }$ is the matrix elements of the quadratic coupling operator in Eq. 6 among the first two eigenstates of ${\hat{H}}_{0}$. The latter states approximately correspond to the parallel and antiparallel orientation of the spin along the magnetic field direction. *E*_a_ is the energy of said states, $\hbar \omega_{\text{ab}}=E_{a}-E_{b}$, and $\bar{n}={\left[\text{exp}\left(\hbar \omega /k_{B}T\right)-1\right]}^{-1}$ is the Bose-Einstein thermal phonon population. Figure 1 provides a graphical representation of this process. A transition between two spin states, e.g., a spin flip, is induced by the simultaneous interaction of the spin with two phonons, where one is absorbed by the spin and the other is emitted. To fulfill energy conservation, introduced by the presence of a Dirac delta in Eq. 9, the energy difference between the two phonons must match the spin energy splitting. However, the energy of the single phonons generally exceeds that of the spin, i.e., $\omega_{\text{ab}}<<\omega_{\alpha }∼\omega_{\beta }$, and the function *G* becomes

$$G\left(\omega_{\text{ba}}∼0,\omega_{\alpha },\omega_{\beta }∼\omega_{\alpha }\right)∼\frac{e^{{\beta \omega }_{\alpha }}}{{\left(e^{{\beta \omega }_{\alpha }}-1\right)}^{2}}$$ (10)

The mathematical form of Eq. 10 is at the core of the phenomenological treatment of two-phonon spin relaxation (vide infra).

The second contribution to two-phonon relaxation, Γ_II_, comes from linear terms in *Q*_α_ and fourth-order perturbation theory and reads

$$\Gamma_{\text{II}}=\frac{\pi }{2\hbar^{2}}\sum_{\alpha \geq \beta }{|T_{\text{ab}}^{\alpha \beta ,+}+T_{\text{ab}}^{\beta \alpha ,-}|}^{2}G\left(\omega_{\text{ab}},\omega_{\alpha },\omega_{\beta }\right)$$ (11)

where

$$T_{\text{ab}}^{\alpha \beta ,\pm }=\sum_{c}\frac{V_{\text{ac}}^{\alpha }V_{\text{cb}}^{\beta }}{E_{c}-E_{b}\pm \hbar \omega_{\beta }}$$ (12)

All the terms in Eqs. 11 and 12 carry the same meaning as in Eq. 8, with the additional presence of a sum over the excited states, *c*. The latter commonly take the name of virtual states to stress that although they correspond to real molecular electronic states, the process described by Eq. 11 does not involve their population and should not be confused with an Orbach process (*18*, *19*). The energy of the states *c* for the systems of interest here exceeds any phonon energy (vide infra), and a resonant Orbach process can be safely excluded. The presence of virtual states in Eq. 11 rather expresses the fact that the lattice perturbs the system to a point where excited states are admixed into the ground state. This relaxation mechanism is graphically described in Fig. 1, where the same spin and phonon transition as before are concerned, but the process is now mediated by virtual transitions.

It is important to note that the choice of working within the framework of the full electronic Hamiltonian or the effective spin Hamiltonian has important implications in spin-1/2 systems. In the case of the use of the effective spin Hamiltonian, only the ground-state Kramers doublet (KD) is present in the model and Eq. 11 cannot be applied due to the lack of any excited state able to promote virtual transitions. Working with the spin Hamiltonian then forces the use of Eq. 8 to simulate two-phonon spin relaxation, while both Γ_I_ and Γ_II_ can in principle be computed in the full electronic Hamiltonian framework.

### Modeling of relaxation

Equations 8 and 11 provide a direct link between the principles of quantum mechanics and spin relaxation time as an experimental observable. We now describe how these equations can be used in practice to make predictions. There are essentially two different philosophies: (i) implement them from first principles or (ii) simplify them further to determine simplified models with a few parameters that can be fit to experiments, i.e., a phenomenological approach.

In the first approach, the Hamiltonian, the phonons, and the coupling coefficients are all determined from first principles on a case-by-case through electronic structure calculations, and used as input to Eqs. 7 to 12. By their own very nature, ab initio simulations describe the molecular crystal’s electronic and vibrational structure starting from nothing more than the total number of electrons and atoms’ positions in the crystal cell. This makes it possible to automatically account for fundamental symmetries of the problem, including the time-reversal symmetry of Kramers’ systems. This approach not only makes it possible to provide an estimate of *T*_1_ without resorting to fitting experimental data but also brings to light some qualitative limitations of the common assumptions around the modeling of these systems. In particular, the Debye model is often used to model the phonons of molecular crystals, although it had been designed as an idealized picture of extended ionic crystal lattices, where a single atom per unit cell is present and acoustic modes fully account for the total vibrational density of states. However, even in the case of Cu(II) in ionic lattices, the real phonon density of states deviates from a simple Debye one. Moreover, the replacement of the Debye model with an experimentally determined phonon distribution is insufficient, by itself, to predict spin-lattice relaxation (*35*–*37*). This limitation is even more severe in molecular crystals of coordination compounds, where the phonon density of states deviates substantially from a Debye model (*26*, *38*–*42*). In particular, very low energy (down to 10 cm^–1^) optical phonons are present in these molecular crystals. This leads to a strong admixture with acoustic modes, for which distinctive linear dispersions remain visible only very close to the Γ-point. In terms of molecular motions, these low-energy phonons are characterized by a mixture of intramolecular distortions and local rotation, which can efficiently modulate spin interactions (*26*) and lead to relaxation.

Accurate ab initio solutions to the problem of spin-phonon relaxation have only become available in recent years (*18*), and the phenomenological approach has been the preferred way to model spin relaxation since Orbach’s proposal to do so. Over the decades, several phenomenological expressions for *T*_1_ have been derived based on Eqs. 8 and 11. Although a plurality of approximations and assumptions have been tested, three of them are recurrent: (i) the low-energy spectrum of the phonons is described by a Debye model, i.e., the phonons’ density of states is a continuous quadratic distribution up to the Debye energy cutoff value $k_{B}\theta_{D}$; (ii) additional dispersion-less phonons, often referred to as local modes, might be available at energies higher than the Debye cutoff; (iii) all phonons couple to the spin in the same way. In addition to this, the time-reversal symmetry has to be included in the model by hand in the case of Kramers’ systems. Under these assumptions, the following expression for *T*_1_ can be derived for two-phonon processes

$$\frac{1}{T_{1}}=A{\left(\frac{T}{\theta_{D}}\right)}^{9}J_{B}\left(\frac{\theta_{D}}{T}\right)+B\frac{e^{\Delta /T}}{{\left(e^{\Delta /T}-1\right)}^{2}}$$ (13)

where

$$J_{B}\left(\frac{\theta_{D}}{T}\right)=\int_{0}^{\theta_{D}/T}x^{8}\frac{e^{x}}{{\left(e^{x}-1\right)}^{2}}\mathrm{dx}$$ (14)

θ_D_ is the Debye temperature in K, Δ is the energy of the local mode in K, and *A* and *B* are parameters that are adjusted to fit the experimental data. The first rhs term in Eq. 13 describes a relaxation process commonly named as Raman and describes a two-phonon process involving phonons with energies below θ_D_. For *T* below θ_D_, the Raman process predicts a temperature dependence of *T*^9^, which gradually changes to *T*^2^ at temperatures well above θ_D_. The local mode (second rhs term in Eq. 13) is also a two-phonon process, but differently from the Raman one, it involves a discrete vibrational energy that is above the Debye temperature. This distinction between Raman and local-mode relaxation is lost in the ab initio approach, as it treats all vibrational degrees of freedom on the same footing, and we generally refer to two-phonon relaxation as a broad term encompassing both mechanisms. Equation 13 has been used to analyze, for example, the temperature dependence of *T*_1_ for Cu(II) complexes (*15*, *43*), vanadyl complexes (*44*, *45*), and V(IV) trischelates (*46*). Reported values of θ_D_ are in the range of 60 to 125 K (*15*, *43*–*46*). Energies of local modes for magnetically dilute spins have been reported in the range of 100 to 300 cm–1 for copper porphyrins (*47*), phthalocyanine (*48*, *49*), chelate complexes (*43*), vanadyl phthalocyanines (*44*, *49*), and V(IV) trischelates (*46*). Although the actual phonon distributions in crystals have been shown to deviate from the Debye model (*35*, *36*), the empirical fitting parameters indicate the range of phonon energies that dominate relaxation.

### Experimental and computational results

**Fig. 2.**
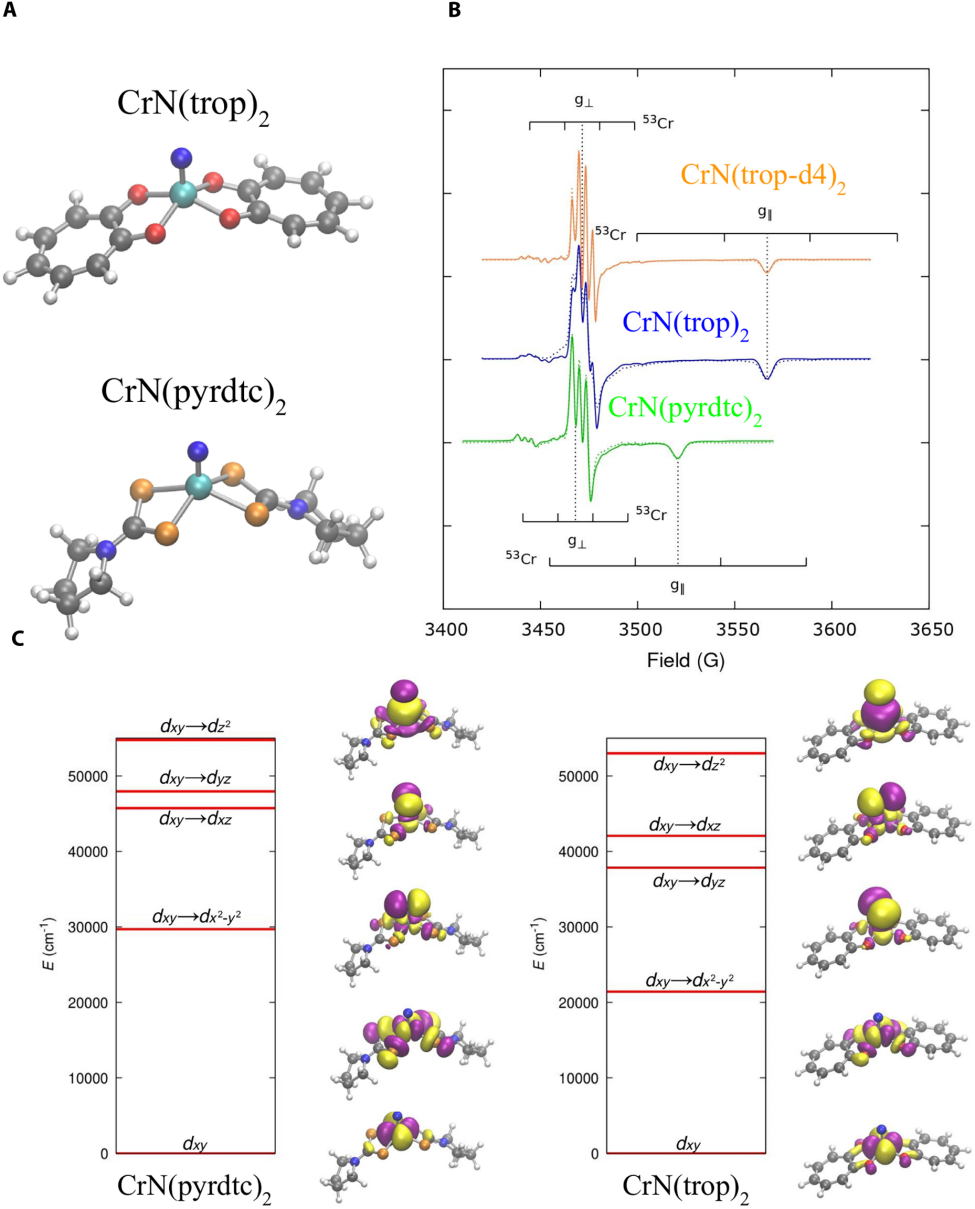
CrN(pyrdtc)_2_ and CrN(tTrop)_2_ electronic properties. (**A**) Molecular structures. Color code: cyan for Cr, blue for N, gray for C, red for O, orange for S, white for H. (**B**) EPR spectra. Simulated (solid line) and measured (dashed line) CW-EPR spectrum for CrN(pyrdtc)_2_ in green, CrN(trop)_2_ in blue, and CrN(trop-d4)_2_ in orange. For the *I* = 0 ^52^Cr, the positions of the *g*_⊥_ and *g*_∥_ positions are indicated with dashed vertical lines. For the *I* = 3/2 ^53^Cr isotope, the hyperfine level positions are marked with black ticks centered around the *I* = 0 values. (**C**) Excited states. NEVPT2 (1,5) excited states for CrN(pyrdtc)_2_ and CrN(trop)_2_. Molecular orbitals for each state are also reported using an isovalue of 0.02 e/Å^3^. Positive (negative) values of the wavefunction are plotted in purple (yellow).

The compounds nitrido bis(pyrrolidine dithiocarbamate)chromium(V) [CrN(pyrdtc)_2_] and nitrido bis(tropolone)chromium(V) [CrN(trop)_2_] are chosen for our investigation of the role of the contributions to two-phonon relaxation in spin-1/2. Their molecular structure is reported in Fig. 2A. Among the many molecules with spin-1/2, Cr(V) systems are particularly advantageous for our study because samples with natural nuclear isotopic abundance are composed of 90.5% ^52^Cr with nuclear spin moment *I* = 0 and 9.5% ^53^Cr with *I* = 3/2, which have overlapping but distinguishable signals in the EPR spectra (see Fig. 2B). This permits the evaluation of the impact of nuclear hyperfine interaction on relaxation in the same sample by selectively measuring at field positions that correspond to transitions for one or the other nuclear isotope. CrN(trop)_2_ and its deuterated counterpart also allow the study of the effect of a rigid auxiliary coordination sphere and nuclear spin-free ligands on spin relaxation time, which have been flagged as possible sources of decoherence (*27*, *50*).

### Synthesis, electronic structure, and magnetism

The synthesis follows the general protocol developed for nitrido-chromium(V) complexes, which involves atom transfer (formally transfer of N^−^) from *N*,*N*′-ethylene bis(salicylideniminato)nitrido manganese(V), MnN(salen), to semi-labile CrCl_3_(THF)_3_ in poorly coordinating solvents. Good yields over 70% are obtained consistently for synthesis carried out in dry acetonitrile. The synthesis of CrN(pyrdtc)_2_ was first reported in (*51*), while CrN(trop)_2_ is new among tropolonate complexes. The solid-state structural parameters determined from single-crystal x-ray diffraction follow similar trends among nitrido complexes. The molecular structures are depicted in Fig. 2A. Structural features and crystal structures are detailed in fig. S1. The complex with deuterated tropolonate [CrN(trop-d4)_2_] is also synthesized following the same procedure. Full deuteration of all positions except the most distant 5-position in the tropolone ring is achieved as previously reported for other derivatives (*52*). Finally, the compound ReN(pyrdtc)_2_, isomorphous to the chromium complex, is synthesized (*53*) to serve as a diamagnetic solid-state host for CrN(pyrdtc)_2_. Dilution of paramagnetic species into a diamagnetic host removes dipolar spin-spin interactions that interfere with the measurement of spin-lattice relaxation.

The electronic excitations for CrN(pyrdtc)_2_ and CrN(trop)_2_ are computed with multireference methods and reported in Fig. 2C together with their description in terms of molecular orbitals, revealing a single unpaired electron located in the *d*_*xy*_ orbital of their Cr(V) ion. Excited states corresponding to intra-band *d-d* transition are present at high energy, starting from ~30,000 cm^−1^ for CrN(pyrdtc)_2_ and ~20,000 cm^−1^ for CrN(trop)_2_, revealing a well-isolated spin-1/2 Kramers doublet ground state. The lowest energy *d-d* transition in the experimental ultraviolet-visible spectrum for CrN(pyrdtc)_2_ is at 18,300 cm^−1^ (*51*, *54*) and at 18,000 cm^−1^ for CrN(trop)_2_ (see fig. S26). Simulations are in better agreement with the experiment for CrN(trop)_2_ than for CrN(pyrdtc)_2_ for which the transition energy is overestimated by about 50%. The reason can be traced back to the underestimation of ligand-metal covalency in our computational approach. A detailed discussion about this is presented in the Supplementary Materials, where we show that larger active spaces in our multi-reference simulations solve this discrepancy but at large computational costs. The role of the accuracy of the spectrum on the spin relaxation simulations is also discussed in detail.

Because of the large energy separation between the ground and excited states, these compounds represent the prototypical systems where their magnetic properties can be accurately described by an effective spin-1/2 Hamiltonian. The X-band continuous wave (CW) spectra of CrN(pyrdtc)_2_, CrN(trop d-4)_2_, and CrN(trop)_2_, reported in Fig. 2B, were interpreted with the spin Hamiltonian in Eq. 4. The dominant features are from the *I* = 0 ^52^Cr isotope, which is about 90.5% of natural abundance. Weaker but well-defined lines are observed for the *I* = 3/2 ^53^Cr isotope (see Fig. 2B). The eigenvalues of **g** and **A** obtained by simulating the EPR spectra are summarized in Table 1 and exhibit characteristic axial anisotropy. The symmetry axis roughly coincides with the CrN chemical bond and will be referred to as the parallel (∥) direction. The average eigenvalues of **g** associated with the eigenvectors lying in the perpendicular direction (*g*_⊥_) for CrN(pyrdtc)_2_ and CrN(trop)_2_ are similar, but the *g*_∥_ value for CrN(trop)_2_ is substantially lower than for CrN(pyrdtc)_2_ due to larger spin-orbit coupling. Comparison with literature values for other Cr(V) nitrido complexes (*17*, *55*) is provided in fig. S4. The eigenvalues of **g** and *A* for CrN(pyrdtc)_2_ in 3:7 CH_2_CL_2_:toluene and doped into the Re analog are the same within experimental uncertainty, demonstrating that the two different environments do not significantly alter the molecular structure.

**Table 1.** Spin Hamiltonian parameters. The table reports the eigenvalues of **A** and **g** from Eq. 4 obtained by fitting the CW-EPR spectra and simulated with DFT and NEVPT2. Hyperfine couplings are reported in MHz. Experimental values for CrN(pyrdtc)_2_ and CrN(trop)_2_ were obtained at 60 to 70 K for the compound diluted in the Re analog and in glassy toluene:CH_2_CL_2_, respectively.

		X-band	DFT	NEVPT2
CrN(pyrdtc)_2_	*g*_iso_	1.9875	1.984	1.983
	*g*_∥_	1.9673	1.969	1.955
	*g*_⊥_	1.9976	1.992	1.996
	*A*_iso_	74.20	79.31	–
	*A*_∥_	–	125.71	–
	*A*_⊥_	49.84	57.33	–
CrN(trop)_2_	*g*_iso_	1.9678	1.976	1.971
	*g*_∥_	1.9415	1.953	1.925
	*g*_⊥_	1.9958	1.986	1.994
	*A*_iso_	74.76	82.61	–
	*A*_∥_	–	128.66	–
	*A*_⊥_	49.84	62.48	–

Table 1 also reports the eigenvalues of the tensors appearing in Eq. 4 calculated by two different electronic structure theory methods. As observed in other studies, density functional theory (DFT) methods are well suited to recover electronic dynamical correlation in systems with a single unpaired *d* electron and achieve a good agreement for both **g** and *A* tensors (*42*, *56*). Simulations performed with NEVPT2 are found to overestimate the *g*_⊥_ shifts but still correctly capture the overall experimental trends. The calculation of **A** at this level of theory has not been attempted, but recent work suggests that this is possible (*57*). In conclusion, both methods adequately describe the electronic structure of these compounds.

### Spin-lattice relaxation measurement

**Fig. 3.**
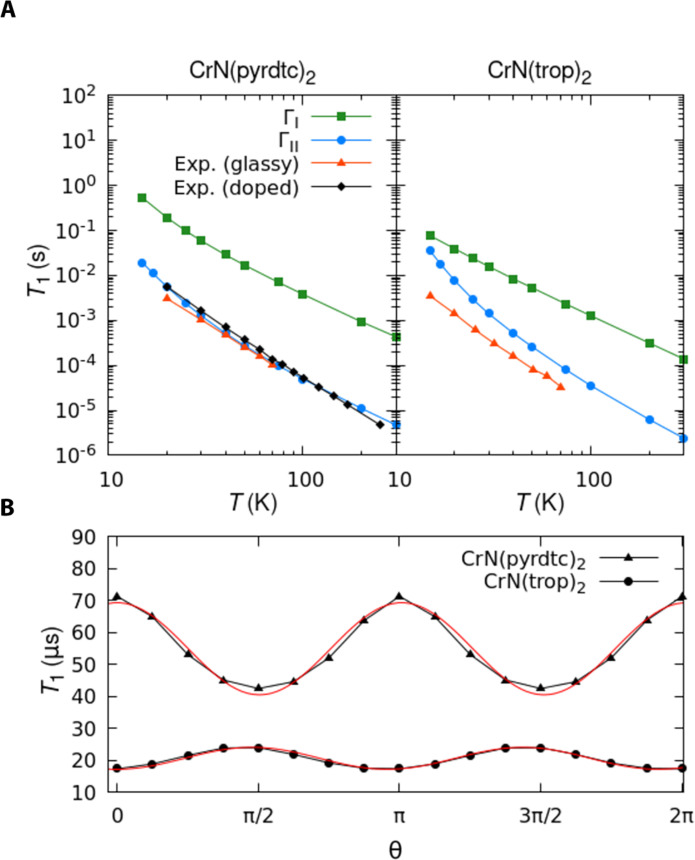
CrN(pyrdtc)_2_ and CrN(trop)_2_ spin-lattice relaxation time. (**A**) Spin-lattice relaxation time for CrN(pyrdtc)_2_ and CrN(trop)_2_. Experimental results obtained with inversion recovery at the *g*_⊥_ field and X-band frequency: Black lines and circles are in doped solid, and red lines and triangles are glassy solution. Blue lines and dots report simulation results obtained with Γ_II_. Green lines and squares report simulation results obtained with Γ_I_. (**B**) *T*_1_ angular dependency. The relaxation time computed at 100 K as a function of the direction of the applied magnetic field is reported. Triangles and circles are points calculated for CrN(pyrdtc)_2_ and CrN(trop)_2_, respectively. Continuous red lines show the fitting of the computed points using *f*(θ) = sin^2^(θ). Comparison with experimental data is provided in the text.

Spin-lattice relaxation for CrN(pyrdtc)_2_ and CrN(trop)_2_ is measured by three-pulse inversion recovery as detailed in Materials and Methods. In these experiments, the orientation of the electron spin is inverted with respect to the direction of the magnetic field, and the time it takes to relax back to the thermal equilibrium, namely, *T*_1_, is monitored. Emphasis is placed on data obtained at temperatures above about 20 K, where two-phonon processes are proposed to dominate (*1*, *58*). Results obtained at the X-band frequency (see Fig. 3A) reveal a sharp decrease of *T*_1_ as a function of increasing temperature, in agreement with the literature on spin-1/2 transition metal complexes (*50*, *55*, *59*, *60*). Faster relaxation for CrN(trop)_2_ than for CrN(pyrdtc)_2_ is consistent with the general trend that compounds with larger *g*-shift (deviation from the free electron value of 2.0023) relax faster due to a larger effective spin-orbit coupling (*12*, *13*, *61*). The differences in *T*_1_ between the two compounds may also be partly attributed to the different host matrix, Re-doped solid and glassy solution, respectively. The latter environment likely presents softer lattice vibrations, which are known to speed up spin relaxation (*12*). However, the similarity between relaxation rates for CrN(pyrdtc)_2_ in the Re-doped solid and glassy solutions (Fig. 3A) suggests that the host does not have a large impact on relaxation for these complexes. Values of *T*_1_ for CrN(trop)_2_ in 3:7 CH_2_CL_2_:toluene are indistinguishable from CrN(trop d-4)_2_ in 3:7 CH_2_CL_2_:toluene-d_8_, which shows that nuclear spins play a negligible role.

Experiments were repeated for values of the external magnetic field that correspond to the parallel and perpendicular orientations of molecules with *I* = 0 and *I* = 3/2, relative to the direction of the external magnetic field. Because of the extensive overlap of transitions for the two isotopes, it was not possible to define the full orientation dependence of *T*_1_ for the glassy or powdered samples. The comparison between experiments conducted at X-band and Q-band conclusively shows that the overall intensity of the static magnetic field up to ∼1.2 T does not substantially affect *T*_1_ for the same orientation between field and molecule. For CrN(pyrdtc)_2_ in the Re analog, the ratio of *T*_1_ for the parallel and perpendicular orientations of the molecule in the magnetic field is 1.4 at X-band and 1.8 at Q-band. The larger ratio at Q-band than at X-band is attributed to greater orientation selection at the higher frequency. In glassy toluene:CH_2_CL_2_, the ratio is 1.2. For CrN(trop)_2_, the ratio of *T*_1_ (parallel/perpendicular) is 1.6 at X-band and 1.5 at Q-band. Finally, the comparison between *T*_1_ for the two Cr isotopes does not reveal any substantial difference, supporting the negligible role of hyperfine interactions in the spin relaxation process for these complexes in rigid matrices, and consistent with previous observations for Cr(V) complexes (*17*).

The experimental *T*_1_ versus *T* curves are interpreted with Eq. 13. As discussed in the Supplementary Materials, the temperature dependence of *T*_1_ for CrN(pyrdtc)_2_ in ReN(pyrdtc)_2_ and for CrN(trop)_2_ in 3:7 CH_2_CL_2_:toluene could be fit equally well with two models: (i) two local modes with energies of 55 and 200 cm^−1^ for CrN(pyrdtc)_2_ and 42 and 145 cm^−1^ for CrN(trop)_2_, or (ii) replacement of the lower energy local modes with a Raman process due to a Debye distribution of phonons with θ_D_ of 70 and 55 cm^−1^ for CrN(pyrdtc)_2_ and CrN(trop)_2_, respectively. Both models indicate that a substantial range of low-energy phonons is required to match the data. The ability to fit the data with two rather different models of the phonon distribution is a caution that a match with the temperature dependence of the experimental data does not constitute proof of the validity of the proposed phonon energy distribution responsible for the relaxation. A comparison of the temperature dependence of *T*_1_ for CrN(pyrdtc)_2_ and CrN(trop)_2_ with other Cr(V) complexes is reported in fig. S4. The substantial similarities in the temperature dependence of *T*_1_ across compounds and lattices suggest that the calculations and interpretations in this paper have important implications for other Cr(V) complexes.

### Spin-lattice relaxation simulations

With the aim of determining the leading contribution to two-phonon relaxation in spin-1/2, we start by using the spin Hamiltonian framework and Eq. 8 to simulate *T*_1_ as a function of temperature, field intensity, and orientation of molecules in the external field. Simulations are compared with the experimental data from inversion recovery experiments. When the separated contributions of **A** and **g** to Eq. 5 are considered, the modulation of **A** is predicted to be the leading relaxation mechanism at all fields investigated in this study (~0.33 and 1.2 T) and to lead to values of *T*_1_ that are independent of the magnitude of the external magnetic field. The modulation of **g** instead leads to $T_{1}\propto B^{-2}$, which is inconsistent with the observed similarity of *T*_1_ values at X-band (~0.33 T) and Q-band (1.2 T). When studying the dependence of *T*_1_ on the orientation of the magnetic field, we observe that either the modulation of **A** or **g** leads to orientation anisotropy (see fig. S15). All these results are in agreement with former reports for several Vanadyl compounds at both X- and Q-band (*27*, *42*). However, differently from previous studies, a relaxation mechanism due to the modulation of **A** in the Cr(V) compounds is inconsistent with the similarity in *T*_1_ for the two nuclear isotopes of Cr (*I* = 0 and *I* = 3/2). Moreover, a large deviation between predictions of *T*_1_ and experiments is observed for the Cr(V) compounds, with the predictions based on Eq. 8 overestimating the *T*_1_ by two orders of magnitude (see Fig. 3A). In conclusion, neither the modulation of **A** or **g** is fully consistent with experimental data.

We now consider the alternative two-phonon mechanism of Eq. 11 by abandoning the effective spin Hamiltonian in favor of using the full electronic Hamiltonian (*62*). As depicted in Fig. 3A, simulated values of *T*_1_ using Eq. 11 are in excellent agreement with experimental values for CrN(pyrdtc)_2_ in both solid and solution phases, with only a small difference in the slope of the curves at the highest temperatures studied. A slightly larger deviation between simulations and experiments is observed for CrN(trop)_2_, which we partly attribute to comparing simulations of crystal phonons and measurements in frozen solution. On this note, we also remark that specific choices in the ab initio methods, e.g., size of the active space, influence the predicted values of *T*_1_ to some degree, making it hard to comment on small deviations from experiments. A full discussion is presented in the Supplementary Materials. Figure S21 also reports the dependence of *T*_1_ as a function of the number of states used to compute the virtual transitions in Eq. 12, showing that the first excited KD has the larger contribution to relaxation. If the time-reversal symmetry is broken by selecting an odd number of excited states, a drastic decrease in *T*_1_ is observed. This is coherent with the analysis of relaxation in Kramers systems already discussed in early literature (*7*). Figure 3B reports the calculated value of *T*_1_ as a function of the angular orientation in the external magnetic field. For CrN(pyrdtc)_2_, the simulated ratio of *T*_1_ in the parallel and perpendicular orientation is 1.7 (Fig. 3B), in good agreement with the experimental ratios of 1.4 at X-band and 1.8 at Q-band. CrN(trop)_2_, the calculation gives a reverse dependence, with longer *T*_1_ for the perpendicular orientation by about a factor of 1.4, which is contrary to the experimental ratios of 1.6 at X-band and 1.5 at Q-band. However, we note that the orientation dependency of *T*_1_ in CrN(trop)_2_ is small in both simulations and experiments, making a direct comparison less straightforward. Simulations also correctly reproduce the sin^2^(θ) orientational dependency of *T*_1_ recently reported in the literature for other spin-1/2 molecules (*16*, *63*). This description of two-phonon spin relaxation is found to be independent of the magnitude of the external magnetic field and the nuclear isotope, therefore fitting all experimental evidence.

**Fig. 4.**
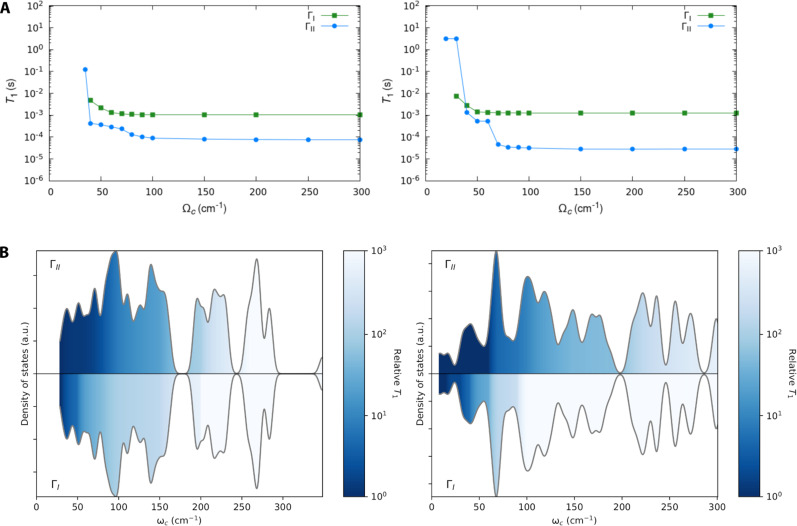
Dependency of *T*_1_ on normal modes. (**A**) High-energy phonon energy cutoff dependence of relaxation time. The value of *T*_1_ at 100 K is reported for CrN(pyrdtc)_2_ (left) and CrN(trop)_2_ (right). *T*_1_ is computed with Γ_I_ (green squares and line) and Γ_II_ (blue squares and line) by excluding phonons with ω > Ω_c_. (**B**) Low-energy phonon cutoff dependence of relaxation time. Phonon density of states is reported for CrN(pyrdtc)_2_ (left) and CrN(trop)_2_ (right). The curves are shaded according to the contribution to the computed relaxation time *T*_1_ at 100 K, with the exclusion of phonons with ω < ω_c_, where the latter corresponds to the value reported on the *x* axis. Coloring of the phonon distributions above the center line and reflected below the center line correspond to the relaxation times obtained with Γ_II_ and Γ_I_, respectively, and normalized to the value obtained including all the phonons in the simulation.

Finally, we address the important question of identifying the relevant phonons for spin relaxation. Figure 4 reports the phonon density of states, where the absence of acoustic modes in our simulations appears as a gap below ∼20 cm^−1^. Although a full integration of the Brillouin zone is always advisable, it comes at a large computational cost and it has been shown previously that a Γ-point approximation does not drastically affect the simulation of two-phonon processes. To determine the most relevant vibrational energy window, the simulation of *T*_1_ at 100 K is performed including all phonons up to a cutoff Ω_c_. The full results of Fig. 3A are recovered for Ω_c_ → ∞. Figure 4A reports the results of this analysis for both compounds, clearly showing that predicted values of *T*_1_ converged by about Ω_c_ ∼ 70 cm^−1^ for both mechanisms considered (Γ_I_ and Γ_II_), meaning that phonons of higher energy do not contribute to the observed values of relaxation time. This result is in agreement with previous considerations based on calculations, where the optical phonons with the lowest energy were identified as the dominant contribution to relaxation (*19*, *27*, *42*). This result is understood by noticing two key facts: (i) The thermal population of phonons, $\bar{n}$, decreases rapidly as a function of their energy for a given *T*, making high-energy phonons less effective (see fig. S25), and (ii) that a large density of states is observed in the low energy part of the vibrational spectrum of the lattice (reported in Fig. 4B). We now turn to the question of how much *T*_1_ would increase if we removed the lowest energy phonons, e.g., by synthetic design. To answer this question, we perform the simulation of *T*_1_ at 100 K including all phonons down to a lower cutoff ω_c_. When ω_c_ is set to a lower value than the first available phonon, the full results of Fig. 3A are recovered. By shifting ω_c_ to higher values, we exclude the low energy phonons from the computation and observe how *T*_1_ is affected. Figure 4B reports the results of this analysis for both compounds. Through the color scale, we can observe that the value of *T*_1_ predicted through Γ_I_ increases drastically as ω_c_ is increased. When we consider the contribution of phonons through Γ_II_, we observe a slightly different scenario, with a slower increase of *T*_1_ as ω_c_ is increased, effectively pointing to the fact that although the lowest energy phonons are still the ones that determine *T*_1_ through a winner-takes-it-all effect, a large window of vibrations provides only slightly less effective alternative relaxation pathways and that phonons up to ~120 cm^−1^ would need to be removed to extend *T*_1_ by one order of magnitude at 100 K. The thermal population of phonons enters the expression of Γ_I_ and Γ_II_ in identical ways and the explanation for such a difference between the two mechanisms must be found in the different prefactors of the function *G*. The expression of Γ_II_ shows that phonons at high energy have increased impact as the denominators of the functions $T_{\text{ab}}^{\alpha \beta ,\pm }$ are decreased. Such a condition is, however, weaker than the one imposed by the phonon thermal population in virtue of the fact that the former is a quadratic expression and the latter is exponential. As a result, phonons with energy higher than the lowest ones offer more efficient alternative relaxation pathways in Γ_II_ than in Γ_I_.

## DISCUSSION

**Fig. 5.**
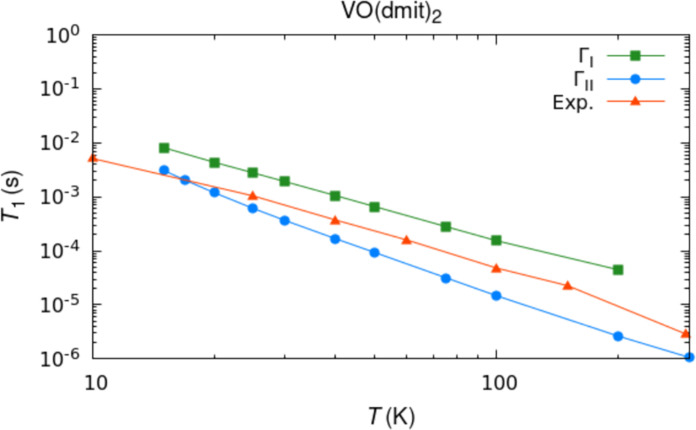
VO(dmit)_2_ spin-lattice relaxation time. Red lines and triangles correspond to experimental results obtained with inversion recovery at X-band frequency. Blue lines and dots report simulation results obtained with Γ_II_. Green lines and squares report simulation results obtained with Γ_I_.

Despite their conceptual simplicity, the ab initio description of spin relaxation in spin-1/2 molecules has proven to be the hardest nut to crack among a variety of magnetic systems (*18*). Early ab initio modeling of *T*_1_ had been based on a spin Hamiltonian ansatz and attempted to quantitatively predict spin relaxation in spin-1/2 in terms of the modulation of the **g** or **A** tensors (*14*, *64*). Both previous (*19*, *26*, *27*) and present computational results have, however, shown that the modulation of the *g*-tensor cannot account for spin relaxation in spin-1/2 molecules, due to its spurious field dependence (*15*, *16*, *65*) and leading to overall too long values of *T*_1_. Without abandoning the conceptual framework of the spin Hamiltonian, the hyperfine interaction stood out as the only interaction able to explain spin relaxation in spin-1/2 systems. The modulation of **A** has adequately explained all experimental evidence in the case of vanadyl complexes (see Fig. 5) (*19*, *27*, *42*). However, for the Cr(V) nitrido complexes, the modulation of **A** is not consistent with values of *T*_1_ that are independent of nuclear spin. At the same time, spin-lattice relaxation times for a variety of organic radicals and transition metal complexes have been observed to decrease as the *g*-shift values increase (*1*, *12*, *13*, *58*, *61*), pointing to a key role of spin-orbit coupling in the process (*16*).

As shown here, the solution to this puzzle can be found by replacing an effective spin Hamiltonian formalism to interpret the dynamical properties of spin systems (*66*). This result harmonizes decades of literature by confirming that Van Vleck’s original theory is the correct description of two-phonon spin relaxation (*10*). In addition, it provides the first ab initio implementation of the said theory for spin-1/2 molecules. The results obtained in this work for CrN(pyrdtc)_2_ and CrN(trop)_2_ also demonstrate the importance of molecular electronic excited states contributing to the virtual transitions described by Γ_II_, dominating two-phonon spin relaxation at a temperature above ~20 K. The theory proposed in this work naturally explains the experimental correlation between the *g*-shift and relaxation time. Spin-orbit coupling is at the same time the origin of the *g*-shift and a fundamental ingredient of ${\hat{H}}_{e}$ entering Eq. 3. An increased value of such interaction leads both to a stronger coupling between lattice and spin and to deviations to the free electron *g*-value.

It is also important to remark that Γ_I_ and Γ_II_ mechanisms are operative at the same time. Depending on the relative strength of the crystal field and hyperfine coupling, either mechanism could become the leading one for a given molecule. To stress the importance of accounting for all the relaxation mechanisms in the interpretation of relaxometry experiments, we show in Fig. 5 the simulation of two-phonon relaxation time for the compound VO(dmit)_2_ (*65*) using both Γ_I_ and Γ_II_. Some of the present authors previously simulated the spin-lattice relaxation time for this molecule based on the Γ_I_ mechanism and found good agreement with experiments (*19*). The inclusion of Γ_II_ contributions shows that although the latter is the dominant mechanism, the predicted relaxation rates are similar. This situation makes it hard to attribute spin relaxation to a single mechanism, especially once numerical uncertainties are considered. The application of the present methods to a larger pool of molecules will be necessary to confirm whether Γ_II_ is invariably the leading relaxation mechanism in spin-1/2 transition metal complexes. As this manuscript was being prepared, Shushkov reported a DFT-based computational method to predict the relaxation rate of Cu(II) spin-1/2 molecules (*34*). Differently from the method presented here, Sushkov’s approach is based on an effective Hamiltonian that includes nonadiabatic terms to Eq. 5 and as such depends on excited state energies. This method stands out as an alternative description of spin relaxation in spin-1/2 to the one provided here, and the equivalence or difference between the two will need further progress on both the theoretical and computational fronts.

Our study also shines light on the identification of the phonons responsible for relaxation. The Debye temperatures found by analysis of the temperature dependence of *T*_1_ using Eq. 13 are 70 cm^−1^ for CrN(pyrdtc)_2_ doped into the Re analog and 55 cm^−1^ for CrN(trop)_2_ in CH_2_CL_2_:toluene. Although the Debye temperatures are lower than the upper limits of the phonon distributions calculated with Γ_II_, both analyses point to an important role of relatively low-energy phonons in driving *T*_1_ in the Raman regime for these compounds. On the other hand, we stress that although phenomenological fitting of relaxation data seems to correctly capture the energy range of the phonons driving Raman relaxation, it provides a qualitatively different interpretation of the relevant phonons’ nature. The Debye expression for Raman relaxation is derived under the assumption of a spectrum solely composed of long-wavelength pseudo-acoustic phonons, while ab initio simulations are able to quantitatively reproduce the complex molecular crystal vibrations observed in inelastic neutron and x-ray scattering experiments (*40*, *42*). These facts, together with the existence of multiple phenomenological expressions able to fit experimental data equally well (see discussion in the Supplementary Materials), make the ab initio interpretation of relaxation much more robust and insightful. The analysis of relaxation data with Eq. 13 also highlights the necessity of including the effect of a local-mode process with energy of 200 and 145 cm^−1^ for CrN(pyrdtc)_2_ and CrN(trop)_2_, respectively. This effect is not very marked in the present compounds, but similar observations of local modes in the range of 100 to 300 cm^−1^ have been reported before for magnetically dilute copper porphyrins (*47*), phthalocyanines (*48*, *49*), chelate complexes (*43*), vanadyl phthalocyanines (*44*, *49*), and V(IV) trischelates (*46*). Ab initio simulations do not seem to capture this contribution. While discrepancies at low *T* can be easily interpreted as the lack of acoustic phonons in the simulations, the high-*T* behavior cannot be easily reconciled with the current theoretical framework. The latter inevitably leads to the high-*T* limit of *T*_1_ ∝ *T*^−2^, thus underestimating the experimental *T*_1_ in the temperature range in which empirical fittings invoke a high-energy local mode. Numerical tests where the coupling to spin of high-energy phonons in the range individuated by the local-mode analysis is artificially modified find that at least a factor of 4 increase in the coupling would be necessary to have a visible effect of the predicted *T*_1_ for the Cr(V) complexes (reported in fig. S18). Although a systematic investigation of the accuracy of electronic structure methods has not been performed yet, successful applications of our method to other compounds (*19*, *20*) make it improbable that numerical inaccuracies could lead to such a drastically different qualitative picture of relaxation. On the other hand, the accuracy of our methods in the prediction of low-energy vibrations in crystals has been thoroughly benchmarked against experiments (*40*, *42*). Further work will be needed to determine whether the present method identifies phonon modes at these high energies for complexes in which local modes have been clearly identified.

Because of these results on the interpretation of two-phonon relaxation, we are now in the position to critically review the list of chemical strategies at our disposal to control *T*_1_. Achieving a large separation among ground and excited states as well as reducing the density of states in the low-energy part of the vibrational spectrum point to longer values of *T*_1_. The different relaxation rates of CrN(pyrdtc)_2_ and CrN(trop)_2_ can be interpreted with the following considerations. The lower energy of the excited state for CrN(trop)_2_ increases the relaxation rate, which may be partially offset by changes in the lower energy phonons. The overall remarkable properties of these square pyramidal complexes can also now be easily interpreted through the presence of the strongly σ and π donor nitrido group, which induces a very large splitting of *d* orbitals. In light of these considerations, one would be tempted to employ heavier elements like 4*d* or 5*d* transition metals, which boast much larger crystal field splitting (*67*). This, however, would also lead to an increased value of effective spin-orbit coupling, which is well documented as being detrimental for *T*_1_ (*28*). Recently, the use of metal *s* orbitals has been proposed as an elegant way to achieve large energy separations between electronic states and minimizing spin-orbit coupling (*61*). Despite having provided a strong reinterpretation of the two-phonon spin relaxation mechanism in spin-1/2 and the justification for its chemical control guidelines, the latter does not drastically shift from those previously inferred from considering the modulation of the *g*-tensor as the leading relaxation mechanism (*14*, *64*). The *g*-shift and the residual orbital angular momenta of a molecule, previously reported to determine *T*_1_ (*14*, *64*, *68*), also depend on the energy of excited states and spin-orbit coupling. Thus both calculations and experiments are consistent with a large crystal field splitting as a design rule for long-lived spin excitations. Similarly, for what concerns the phonons, both two-phonon mechanisms have the largest contribution from low-energy vibrations, making previous advice still valid (*42*), albeit with reduced expected effectiveness, as suggested by Fig. 4.

In conclusion, our theoretical and experimental study has demonstrated that the interpretation of spin relaxation in spin-1/2 compounds requires going beyond considering only the ground state of molecules and including the impact of high-energy electronic states. The inclusion of these contributions not only made it possible to reproduce experiments to an unprecedented level of accuracy but also provided improved conceptual tools to study and chemically engineer this important class of compounds. We envision that further development of ab initio open quantum system theory in synergy with novel experiments will soon make it possible to achieve a long-sought full reconciliation between theory and experiments.

## MATERIALS AND METHODS

### Syntheses and sample preparation

All solvents were high-performance liquid chromatography (HPLC) grade from VWR Chemicals, except for dichloromethane (DCM), which was from Fischer Chemical. Mn(N)salen and mer-Cr(CL)_3_(THF)_3_ were synthesized as previously reported (*51*). Tropolone (98%) was obtained commercially from Combi Blocks and recrystallized from isopropanol before use. CF_3_COD (Sigma-Aldrich), platinum on carbon, 10% (Sigma-Aldrich), and ammonium pyrrolidine dithiocarbamate (Acros) were used as received. CrN(pyrdtc)_2_ and RenCl_2_(PPh_3_)_2_ were synthesized as described in the literature (*51*, *53*).

#### Synthesis of tropolone-d4

The deuteration of tropolone followed literature protocols for aromatic compounds. Tropolone (1 g; 8.19 mmol) and 10% Pt/C (0.2 g) were added to deuterium oxide (15 g; 75 mmol) (*52*). The reaction was kept acidic by addition of two drops of d-trifluoroacetic acid to subdue rearrangement of the tropolonate under the forcing conditions. The reaction was done in a Teflon-lined steel autoclave at 145°C for 3 days. Aqueous workup and recrystallization from isopropanol yielded 0.390 g (37.8%) of tropolone-d4. The deuteration degree was inferred to be 80% from the high-resolution mass spectrometry of the resulting Cr(N)(trop-d4)_2_, in agreement with the previously reported difference in reactivity of the 5-position compared to the remaining ring positions (*52*).

#### Synthesis of Cr(N)(trop)_2_ and Cr(N)(trop-d4)_2_

The procedure follows the general procedure described by one of the authors (*51*). mer-Cr(CL)_3_(THF)_3_ (0.153 g; 0.409 mmol) and Mn(N)(salen) (0.275 g; 0.821 mmol) were mixed in acetonitrile (10 ml) under a nitrogen atmosphere. The reaction was stirred at room temperature for 20 min, and the precipitated Mn(Cl)(salen) was filtered off. To the filtrate, tropolone (0.105 g; 0.859 mmol) in acetonitrile (3 ml) was added dropwise while stirring under N_2_ upon formation of a brownish orange precipitate. After completion of the precipitated, the product was filtered off and washed with acetonitrile (two times, 30 ml) before being dried in a stream of N_2_. The yield was 89.5 mg (71.1%) of a brownish orange microcrystalline product. The polycrystalline sample was void of Cr(trop)_3_ based on the comparison with the powder x-ray diffraction (Cu-Kα) of the latter. Crystals suitable for single-crystal x-ray diffraction were obtained by recrystallization from acetonitrile/C_2_H_2_ (1:2). The deuterated version Cr(N)(trop-d4)_2_ was synthesized by the same protocol, yielding 70.5% of isolated product with similar peak positions in the powder x-ray diffraction as the nondeuterated sample. High-resolution mass spectrometry, mass/charge ratio (*m*/*z*) = 317.0604 (100%), 316.0540 (92%) corresponding to >80% deuteration.

#### Synthesis of ReN(pyrdtc)_2_ and nitridobis (pyrrolidinedithiocarbamato)rhenium(V)

ReNCL_2_(PPh_3_)_2_ (0.60 g, 0.75 mmol) and NH_4_(pyrdtc) (0.50 g, 3.0 mmol) were stirred in acetone (60 ml) overnight at 40°C under N_2_. The brownish solution containing a yellow precipitate was evaporated to dryness in a stream of N_2_. The resulting solid was extracted with DCM/MeOH (9:1, 100 ml) and filtered, and the extract was reduced in volume to ~10 ml, yielding a bright yellow crystalline product, which was washed with ether, and dried in vacuum (0.24 g, 48%). Crystals suitable for single-crystal x-ray diffraction were obtained by slow evaporation of a DCM solution.

#### X-ray structure determination

Single crystals were obtained as described under the synthesis. They were coated with mineral oil, mounted on kapton loops, and transferred to the nitrogen cold stream of the diffractometer. Diffraction data were recorded at 100(2) K on a Bruker D8 VENTURE diffractometer equipped with a Mo Kα high-brilliance IμS radiation source (λ = 0.71073 Å), a multilayer x-ray mirror and a PHOTON 100 CMOS detector, and an Oxford Cryosystems low-temperature device. The instrument was controlled with the APEX3 software package using SAINT. Final cell constants were obtained from least-squares fits of several thousand strong reflections. Intensity data were corrected for absorption using intensities of redundant reflections with the program SADABS. The structures were solved in Olex2 using SHELXT and refined using SHELXL. Experimental, structure solution and refinement parameters are summarized in table S1.

### Electron paramagnetic resonance spectroscopy

Solutions of 0.6 mM Cr(N)(pyrdtc)_2_ and 0.5 mM CrN(trop)_2_ were prepared by dissolution in CH_2_Cl_2_ followed by dilution with toluene to achieve a 3:7 CH_2_Cl_2_:toluene ratio. A solution of 0.5 mM CrN(trop-d4)_2_ was prepared analogously in 3:7 CD_2_Cl_2_:toluene-d_8_. Approximately 200 μl of solution or the doped ~0.05% Cr(N)(pyrdtc)_2_ in ReN(pyrdtc)_2_ solid was placed in 4-mm outer diameter quartz tubes. Solutions were deoxygenated by multiple freeze-pump-thaw cycles. All EPR samples were backfilled with ~200 mtorr of helium before flame sealing the tube. The low-pressure He provides thermal contact between the sample and the walls of the tube to facilitate thermal equilibration.

EPR spectroscopy and electron spin relaxation time measurements were performed on a Bruker E580 spectrometer with an Oxford CF935 cryostat. X-band data were obtained with an ER4118X-MD5 split ring resonator, and Q-band data were obtained with an ER5107 resonator. For pulse measurements, the X-band resonator was over-coupled to a ringdown 1/e time of ~2.5 to 3 ns, which corresponds to *Q* ∼ 150. The Q-band resonator was overcoupled to permit signal acquisition with less than 200-ns dead time. Temperature was controlled with a ColdEdge/Bruker Stinger closed-cycle helium cooling system. Temperature was monitored with a calibrated Lakeshore Cernox sensor and an Oxford Mercury iTC readout. CW spectra were measured at 60 or 70 K using low microwave power and 0.5 G modulation amplitude at 10 kHz to minimize passage effects. The spectra were simulated using the Bruker software Anisospinfit. *T*_1_ was measured by three-pulse inversion recovery with sequence π − *t* − π/2 − τ − π 2212; τ − echo, with two-step phase cycling, and the value of *t* was incremented to record the recovery curve. The constant τ was set to 360 or 380 ns. The shot repetition time (SRT) was about 10 times the value of *T*_1_ calculated with a stretched exponential. For the relatively high spin concentration in the 0.05% CrN(pyrdtc)_2_ in the Re analog, the time constants were dependent on lengths of the π/2 pulses between 16 and 160 ns. To minimize the impact of spectral diffusion on *T*_1_, the inversion recovery data for 0.05% Cr(N)(pyrdtc)_2_ were acquired with π/2 pulse lengths of 16 ns. For 0.6 mM Cr(N)(pyrdtc)_2_ in 3:7 CH_2_Cl_2_:toluene, the values of *T*_1_ were independent of pulse length, but for consistency with the doped solid, the same pulse lengths were used. For 0.5 mM CrN(trop)_2_ and CrN(trop-d4)_2_ in 3:7 CH_2_Cl_2_:toluene or 3:7 CD_2_Cl_2_:toluene-d_8_, π/2 pulse lengths of 40 ns were used. Inversion recovery curves were not single exponentials, indicating the presence of distributions of relaxation times, which can be represented as a stretched exponential (*69*)

$$Y\left(t\right)=e^{-{\left(t/T_{1}\right)}^{\beta }}$$ (15)

Values of β ranged from about 0.65 at 10 K to about 0.95 at 70 K. All fitted values are reported in tables S2 and S3.

### Electronic structure simulations

Starting from x-ray structures of CrN(pyrdtc)_2_ and CrN(trop)_2_, cell and geometry optimization and simulations of Γ-point phonons have been performed with periodic DFT (pDFT) using the software CP2K (*70*). Cell optimization was performed using a very tight force convergence criterion of 10^−7^ atomic units (a.u.) and Self-Consistent Field (SCF) convergence criteria of 10^−10^ a.u. for the energy. A plane wave cutoff of 1000 Ry, DZVP-MOLOPT Gaussian basis sets, and Goedecker-Tetter Hutter pseudopotentials were used for all atoms. The Perdew-Burke-Ernzerhof (PBE) functional (*71*) and DFT-D3 dispersion corrections (*72*) were used. Phonons were computed with a two-step numerical differentiation of forces and step 0.1 Å.

Electronic structure and magnetic properties were computed on the pDFT-optimized structures using the software ORCA v. 5 (*73*). The decontracted basis set DKH-def2-TZVPP, along with the Douglas-Kroll-Hess (DKH) scalar relativistic correction to the electronic Hamiltonian, was used. Picture-change effects were included in the calculations. The computation of **g** and **A** tensors at the DFT level of theory was performed using a PBE0 exchange-correlation functional (*74*) with the RIJCOSX approximation for Coulomb and Hartree-Fock exchange. Multireference calculations were performed using Complete Active Space Self Consistent Field (CASSCF) in combination with N-Electron Valence Perturbation Theory (NEVPT2). The active space used to build the CASSCF wavefunction is (1,5), i.e., one electron in five 3*d* orbitals. All possible states with multiplicity two were included in the state-average procedure. Mean-field spin-orbit coupling operator, along with quasi-degenerate perturbation theory (QDPT), was used to account for the mixing of spin-free states. Tests with different basis sets and larger active spaces were performed and are reported in the Supplementary Materials.

### Spin-phonon relaxation simulations

First-order vibronic coupling matrix elements of Eq. 3 have been evaluated within the framework described before (*62*). Briefly, starting from the ab initio CASSCF wavefunctions ∣φ_a_〉 and energies *E*_a_, the matrix elements of the $\nabla {\hat{H}}_{e}$ operator are expressed using the Hellmann-Feynman theorem as

$$\langle \varphi_{a}|\nabla {\hat{H}}_{e}|\varphi_{b}\rangle =\nabla E_{b}\delta_{\text{ab}}+\left(E_{b}-E_{a}\right)\langle \varphi_{a}|\nabla \varphi_{b}\rangle$$ (16)

To evaluate the nonadiabatic coupling (NAC) terms 〈φ_a_ ∣∇φ_b_〉, numerical differentiation of wavefunction overlap has been performed using an in-house Python code interfaced with the program WFOVERLAP (*75*). The step for the numerical differentiation has been set to 0.001 Å along the Cartesian coordinates of the system. A systematic convergence study of the *T*_1_ relaxation time as a function of the differentiation step can be found in fig. S17. The evaluation of the second-order terms of Eq. 5 requires computing the second-order derivatives of the tensors **A** and **g** with respect to the molecular Cartesian coordinates. Here, we used a machine learning (ML)–based strategy to calculate the second-order derivatives of the spin Hamiltonian in Eq. 4 (*27*, *42*), as detailed in the Supplementary Materials. We stress that these ML models are merely used to interpolate ab initio data and therefore do not introduce any dependence on experimental data or adjustable coefficients. Once the linear and quadratic derivatives of ${\hat{H}}_{\text{el}}$ and ${\hat{H}}_{s}$ with respect to the Cartesian coordinates, *R*_a_, have been computed, they are transformed into the normal mode reference framework by using

$$\left(\frac{\partial {\hat{H}}_{e}}{\partial Q_{\alpha }}\right)=\sum_{a}^{3N}\sqrt{\frac{\hbar }{\omega_{\alpha }m_{a}}}L_{\alpha a}\left(\frac{\partial {\hat{H}}_{e}}{\partial R_{a}}\right)$$ (17)

and

$$\left(\frac{\partial^{2}{\hat{H}}_{s}}{\partial Q_{\alpha }\partial Q_{\beta }}\right)=\sum_{a,b}^{3N}\sqrt{\frac{\hbar }{\omega_{\alpha }m_{a}}}\sqrt{\frac{\hbar }{\omega_{\beta }m_{b}}}L_{\alpha a}L_{\beta b}\left(\frac{\partial^{2}{\hat{H}}_{s}}{\partial R_{a}\partial R_{b}}\right)$$ (18)

where *m*_*i*_ is the *i*th atomic mass and *L*_αi_ is the Hessian matrix eigenvectors. The vibronic coupling vectors from CASSCF, together with NEVPT2 energies in Eq. 12, are finally used to simulate the contribution of Γ_II_ to spin relaxation by direct calculation of Eqs. 7 and  11. The use of CASSCF vibronic coupling, in conjunction with NEVPT2 vertical excitation energies, is necessary here, as orbital relaxation at the NEVPT2 level is not supported in ORCA v. 5. We also note that the use of a diagonal approximation to the study of the density matrix time evolution, i.e., only considering population terms, is not an issue here as simulations are performed in the presence of an external field and no degeneracies are present (*19*). The ML-predicted second-order derivatives of **g** and **A** are subsequently used to simulate the time dependency of the *z* component of the simulated magnetization $\vec{M}$. Finally, *T*_1_ is extracted for Γ_I_ by fitting *M*_*z*_ with a double exponential function. Further details regarding this approach are provided in the Supplementary Materials. The Dirac delta functions appearing in the spin dynamics equations are broadened with a Gaussian smearing of 30 cm^−1^ after a convergence study on the computed *T*_1_ (see fig. S16). Calculations of relaxation rates and magnetization time evolution were obtained with the development branch T4 of the software MolForge (*19*), available at github.com/LunghiGroup/MolForge.
