# Supplementary material for: The role of electronic excited states in the spin-lattice relaxation of spin-1/2 molecules
Source: Sci Adv. 2025 Feb 12;11(7):eadr0168. doi: 10.1126/sciadv.adr0168 (PMC11817928; doi:10.1126/sciadv.adr0168)
Supplement: Supplementary file 1 — Supplementary Text Figs. S1 to S26 Tables S1 to S22 [file sciadv.adr0168_sm.pdf]

Science Advances
AAAS

Supplementary Materials for

The role of electronic excited states in the spin-lattice relaxation of spin-1/2 molecules

Lorenzo A. Mariano *et al.*

Corresponding author: Sandra S. Eaton, sandra.eaton@du.edu; Alessandro Lunghi, lunghia@tcd.ie

Sci. Adv. **11**, eadr0168 (2025)
DOI: 10.1126/sciadv.adr0168

This PDF file includes:

Supplementary Text
Figs. S1 to S26
Tables S1 to S22

Modeling the Temperature Dependence of $1/T_1$

Eq. 13 and 14 in the main text were used to model the temperature dependence of T_1 (see Table S4 and Fig. S3).

Very similar fits to the experimental data were obtained with either of two models – two local modes or a Raman process and a local mode. The parameters for the two models are in Table S4. The plots in Figure S3 below are for two local modes. The ability to fit the data with either model is a reminder that from the perspective of this modelling it is not possible to distinguish between the temperature dependence of $1/T_1$ predicted for a Debye distribution of phonon modes and a single mode with energy slightly below the Debye temperature.

A characteristic feature of the Raman process or the lower energy local mode is that above about 60 K the slope of the plot of $log(1/T_1)$ vs. $log\ T$ becomes less steep than the experimental data. To match the experimental data additional phonons with higher energy are needed, which are modeled as a higher energy local mode. This effect is more conspicuous in the data for $CrN(pyrdtc)_2$ in the Re analog which extend to higher temperatures than could be obtained for $CrN(trop)_2$ in CH_2Cl_2:toluene because of the melting of the glass.

There is a striking similarity in the temperature dependence of the relaxation rates for four Cr(V) complexes despite the differences in the solvents or lattice used (Fig. S4). If a Raman process with a relatively low Debye temperature or a local mode with relatively low energy dominated the relaxation the slope of the plot would become smaller at higher temperatures. To model the observed temperature dependence requires a relatively broad range of phonon energies.

Neural network interpolation of spin Hamiltonian

The numerical calculation of second-order derivatives of spin Hamiltonian parameters is a heavy computational task as it requires a minimum of about $4N(N+1)$ ab initio simulations, where N is the number of atoms in the molecule. To overcome these prohibitive overheads we use a numerical strategy based on i) training a neural network to interpolate **A/g** as a function of molecular Cartesian coordinates, ii) numerical differentiation of these parameters with the neural network.

Different neural network architectures (with a varying number of hidden layers and nodes) were tested to obtain the most suitable models for **A/g** tensor of $CrN(pyrdtc)_2$ and $CrN(trop)_2$ (Fig. S5). All models were created using Tensorflow and Keras API. They all consisted of an input layer of 3N nodes, where N is the number of atoms in each molecule, and an output layer of 9 nodes, which corresponds to the Cartesian tensor components of the **A/g** tensor. They were each trained separately on a dataset of 2000 randomly distorted structures. The four models with the lowest RMSE on the test set for **A/g** tensor for $CrN(pyrdtc)_2$ and $CrN(trop)_2$ were then selected to perform the following studies on first- and second-order derivatives. Each model was trained on 1600 data points and tested on 200 data points that the model had not seen before. The hyperparameters of each model were optimized using a set of 200 validation data points.

The first- and second-order derivatives of each element of the **A/g** tensors are calculated numerically from the **A** and **g** tensors computed using machine learning. A grid of 6×6×6 points is employed to calculate each derivative, which is then fitted using a 2D second-order polynomial expression through the linear least squares method. A selection of these parameters computed with neural networks are benchmarked against ab initio methods as reported in Fig. S6-13.

These derivatives are subsequently used in Eq. 8 to simulate the spin relaxation process and to calculate the spin-phonon relaxation times T_1. The latter is extracted by fitting the decay of the z-component of the magnetization Mz with a double exponential in the case of the hyperfine-coupling tensor **A**, and with a single exponential for the **g** tensor. The simulated values of M_z used for the fitting are shown in Fig. S14.

Ab initio simulations and electronic structure

Different levels of theory were tested to assess the overall accuracy of the relaxation simulations. The correlation-consistent basis set family cc-pVNZ-DK (N = D, T, Q) was employed since it guarantees convergence to the complete basis set limit for increasing values of N. First, we performed a systematic convergence test on the computed T_1. The results obtained for CrN(pyrdtc)$_2$ at NEVPT2(1,5) level of theory are reported in Fig. S20 (a) and compared with those obtained using DKH-def2-TZVPP. Deviations in the computed T_1 with different basis sets remain well below the typical errors of about one order of magnitude generally associated with *ab initio* spin-relaxation simulations [20].

A study of the vibronic coupling matrix elements ∇H_{ij} for increasing basis set size is also reported in Fig. S20 (b, c, d). The variation in these elements with increasingly large basis sets provides a good estimate of the potential impact of Pulay forces that may arise in the presented scheme due to the use of the Hellmann-Feynman theorem with incomplete basis sets. The minimal deviations in ∇H_{ij} matrix elements as we approach the complete basis set limit, along with the small deviations in the computed T_1, support the assumption that Pulay forces do not significantly impact the results presented in this work.

The impact of expanding the CASSCF active space was investigated. Beginning with an active space containing one electron in five 3d orbitals, or (1,5), additional ligand orbitals with significant overlap with these 3d orbitals were included, resulting in a (9,9) active space with nine electrons in nine orbitals. This expanded active space incorporates the doubly occupied $\pi_{x,y}$ orbitals, arising from the overlap of $p_{x,y}$ orbitals on coordinated nitrogen with Cr $d_{xz,yz}$ orbitals; the doubly occupied σ_z orbital from the overlap between the p_z orbital of coordinated nitrogen and the Cr d_z orbital; and the doubly occupied σ_{xy} orbital, formed by the interaction of Cr's $d_{x^2-y^2}$ orbital with the ligand in the plane. These orbitals are plotted in Fig. S23 and S24. To assess the quality of the calculations, the results are compared with two observables: the experimental UV-VIS spectra and the measured g-values. Values of UV-VIS for the CrN(pyrdtc)$_2$ compound are taken from Ref. [51], while the spectrum for CrN(trop)$_2$ was obtained for a CH_2Cl_2 solution with a Perkin Elmer Lambda 2 spectrometer (Fig. S26). For this latter case, only the first absorption peak was resolved since higher energy transitions were obscured by very intense bands. Tables S5-S16 summarize the results for the excitation energies, while Tables S17-S22 present the results for the computed g-values. Significant differences are observed in both the computed UV-VIS spectra and g-values, depending on the choice of active spaces and the inclusion of perturbative treatments of dynamical correlations through NEVPT2 theory. Overall, the use of CAS(1,5) underestimates the excitation energies, whereas NEV(1,5) tends to overestimate them. Moving from an active space (1,5) to a (9,9) alters the nature of all excitations except for the first, which retains a $d_{xy} \rightarrow d_{x2-y2}$ character (see first column of tables S5-S16). At this level of theory, both CAS(9,9) and NEV(9,9) negligibly overestimate the excitation energies. It is important to note that the relaxation process is primarily influenced by the first excited Kramers doublet (see Fig. S21), so variations in the first excitation will impact the results of the simulations when comparing different methods. Regarding the simulated g-values, all methods generally overestimate the

g-shift compared with experiments, with NEV(1,5) achieving the best agreement for both compounds. Simulations of T_1 using different methods are presented in Fig. S22. When NEV(9,9) is considered, a faster relaxation is observed compared to NEV(1,5). This difference between the two approaches can be partially attributed to the distinct energy spectra; specifically, the larger energy spacing between the levels in NEV(1,5) slows down the relaxation relative to NEV(9,9). To highlight this effect, we performed simulations using NEV(1,5) vibronic couplings along with NEV(9,9) energies (denoted as NEV(1,5)* in Fig. S22), and NEV(9,9) vibronic couplings with NEV(1,5) energies (denoted as NEV(9,9)* in Fig. S22). For $CrN(pyrdtc)_2$, this substitution has a more pronounced effect on the computed T_1 compared to $CrN(trop)_2$. This is a direct consequence of the larger difference in excitation energies between NEV(1,5) and NEV(9,9) for $CrN(pyrdtc)_2$, as opposed to $CrN(trop)_2$. Finally, it is important to note that none of the investigated methods can achieve good agreement with both the UV-VIS spectra and g-values simultaneously. In principle, these two quantities are related, and accurate predictions of g-values are a direct consequence of properly evaluating excitation energies and spin-orbit coupling elements. Variations in the accuracies of the two computed quantities may be attributed to error cancellation between vertical energies and the composition of the wavefunction. A further expansion of the active space could help mitigate these errors, resulting in better agreement with experimental data, but it comes at the cost of a dramatic increase in the required computational resources. Ultimately, despite the different methods yielding different computed T_1 values, there are no ambiguities regarding the primary relaxation mechanism in these systems.

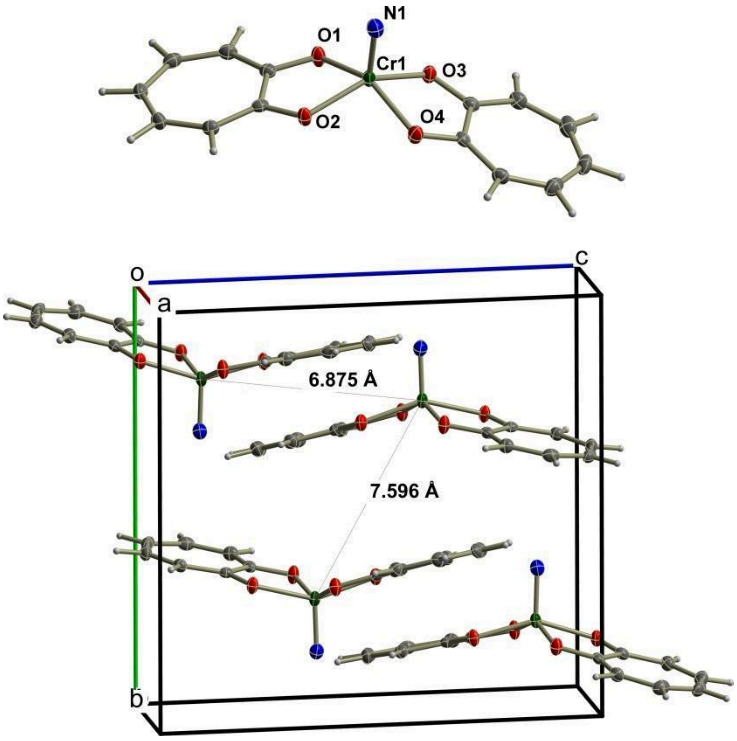

Fig. S1. X-ray Crystallography data for Cr(N)(trop)$_2$.
Top: molecular structure of Cr(N)(trop)$_2$. Bottom: packing with closest metal distances indicated.

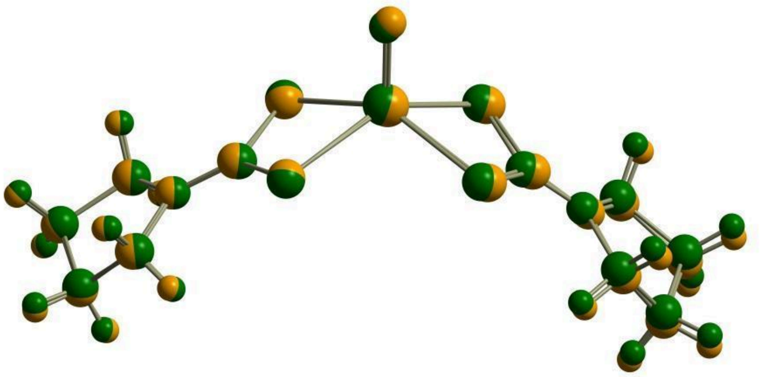

Fig. S2. Overlay of M(N)(pyrdtc)$_2$.

M=Re(orange) and M=Cr(green). Cr-N: 1.549(2) Å, Re-N: Å; Cr-S_{ave}: 2.3777(4) Å; Re-S_{ave}: 2.399(1) Å; N-Cr-S_{ave}: 107.76(5)^o; N-Re-S_{ave}: 107.5(1)^o.

	CrN(pyrdtc)$_2$	CrN(trop)$_2$
CCDC dep. code	CCDC-2181505	CCDC-2352290
Chemical formula	$C_{10}H_{16}N_3ReS_4$	$C_{14}H_{10}CrNO_4$
M_r	492.70	308.23
Crystal system, space group	Monoclinic, $P2_1/c$	Monoclinic, $P2_1/c$
Temperature (K)	120	100
a, *b*, *c* (Å)	14.9044 (11), 8.6125 (6), 11.8925 (9)	7.1912 (8), 12.4977 (16), 13.6290 (18)
β (°)	107.377 (3)	101.376 (4)
V (Å^3)	1456.90 (19)	1200.8 (3)
Z	4	4
Radiation type	Mo *K*α	Mo *K*α
μ (mm^{-1})	8.90	0.97
Crystal size (mm)	0.28 × 0.18 × 0.03	0.19 × 0.17 × 0.08
Data collection		
Diffractometer	Bruker *APEX*-II CCD	Bruker *APEX*-II CCD
Absorption correction	Multi-scan *SADABS2016*/2 (Bruker,2016/2) was used for absorption correction. wR_2 (int) was 0.1337 before and 0.0778 after correction.	Multi-scan *SADABS2016*/2 (Bruker,2016/2) was used for absorption correction. wR_2(int) was 0.1095 before and 0.0722 after correction.
T_{min}, T_{max}	0.390, 0.747	0.668, 0.747
No. of measured, independent and observed [$I > 2\sigma(I)$] reflections	50251, 4633, 3915	27110, 3331, 2494
R_{int}	0.051	0.069
$(\sin \theta/\lambda)_{max}$ (Å^{-1})	0.724	0.694
Refinement		
$R[F^2 > 2\sigma(F^2)]$, $wR(F^2)$, S	0.024, 0.052, 1.13	0.039, 0.086, 1.05
No. of reflections	4633	3331
No. of parameters	163	181
H-atom treatment	H-atom parameters constrained	H-atom parameters constrained
$\Delta\rho_{max}$, $\Delta\rho_{min}$ (e Å^{-3})	1.67, −2.69	0.44, −0.53

Table S1. **Experimental SCXRD details.**

A)

	$g_\perp$ (I = 3/2)		$g_\perp$ (I = 0)		$g_{\|}$ (I = 0)		$g_{\|}$ (I = 3/2)	
T (K)	T_1(s)	beta	T_1(s)	beta	T_1(s)	beta	T_1(s)	beta
9.9	1.50E-02	0.74	1.69E-02	0.73	1.88E-02	0.7		
15	3.50E-03	0.81	3.70E-03	0.8	4.80E-03	0.83		
20	1.51E-03	0.86	1.42E-03	0.87	1.42E-03	0.87	1.22E-03	0.83
25.5	6.17E-04	0.87	6.46E-04	0.88	8.91E-04	0.83		
32	3.08E-04	0.89	3.08E-04	0.86	4.95E-04	0.89		
40	1.73E-04	0.94	1.64E-04	0.94	2.41E-04	0.89		
51	8.10E-05	0.91	8.69E-05	0.91	1.55E-04	0.88		
60	5.60E-05	0.95	5.30E-05	0.95	9.10E-05	0.92	9.59E-05	0.9
70.7	3.30E-05	0.91	3.70E-05	0.91				

B)

	$g_\perp$ (I = 3/2)		$g_\perp$ (I = 0)		$g_{\|}$ (I = 0)	
T (K)	T_1(s)	beta	T_1(s)	beta	T_1(s)	beta
20	1.01E-03	0.84	1.05E-03	0.88	1.28E-03	0.84
40	1.58E-04	0.93	1.67E-04	0.93	2.49E-04	0.9
60	4.77E-05	0.95	4.96E-05	0.92	8.51E-05	0.95
71	2.96E-05	0.95	3.11E-05	0.96	5.48E-05	0.9

C)

	$g_\perp$ (I = 3/2)		$g_\perp$(I = 0)		g-par(I = 0)		g-par (I = 3/2)	
T (K)	T_1(s)	beta	T_1(s)	beta	T_1(s)	beta	T_1(s)	Beta
20	1.54E-03	0.86	1.53E-03	0.87	1.92E-03	0.87	2.60E-03	0.88
40	1.80E-04	0.93	1.78E-04	0.93	2.41E-04	0.91	2.66E-04	0.87
60	5.75E-05	0.96	5.75E-05	0.95	1.23E-04	0.91	9.40E-05	0.88

Table S2. Experimental inversion recovery data for $CrN(trop)_2$.
In CH_2Cl_2-toluene at X-band (**A**); in CH_2Cl_2-toluene at Q-band (**B**); in deuterated sample in CD_2Cl_2-tol-d_8 at X-band (**C**). The experimental inversion recovery data were fitted with a stretched exponential, Eq. 8 in the main text.

A)

	$g_\perp$ (I = 3/2)		$g_\perp$ (I = 0)		g-par (I = 0)		g-par(I = 3/2)	
T (K)	T_1 (s)	beta	T_1 (s)	beta	T_1 (s)	beta	T_1 (s)	beta
10	1.25E-01	0.59	2.55E-02	0.53				
20	9.00E-03	0.84	5.70E-03	0.77	7.80E-03	0.79	8.40E-03	0.75
30	2.00E-03	0.72	1.64E-03	0.9				
40	7.88E-04	0.95	6.94E-04	0.94				
50	3.49E-04	0.94	3.71E-04	0.96				
60	2.43E-04	0.95	2.25E-04	0.97				
70	1.55E-04	0.93	1.35E-04	0.92	1.95E-04	0.92	1.65E-04	0.82
79	1.15E-04	0.96	1.07E-04	0.99				
90	7.12E-05	0.95	7.19E-05	0.98				
101	4.44E-05	0.92	5.28E-05	0.98				
121	2.38E-05	0.88	3.25E-05	0.99				
144	1.94E-05	0.96	2.08E-05	1				
170	1.39E-05	0.97	1.33E-05	0.99				
248	5.20E-06	0.96	4.80E-06	0.99				

B)

	$g_\perp$ (I = 3/2)		$g_\perp$ (I = 0)		$g_{\parallel}$ (I = 0)		$g_{\parallel}$ (I = 3/2)	
T (K)	T_1	beta	T_1	beta	T_1	beta	T_1	beta
10	2.82E-02	0.63	2.14E-02	0.71				
20	3.20E-03	0.77	3.08E-03	0.8	3.70E-03	0.78	3.10E-03	0.74
30	1.06E-03	0.88	1.04E-03	0.91				
40	4.88E-04	0.92	4.74E-04	0.95				
50	2.68E-04	0.93	2.59E-04	0.96				
60	1.65E-04	0.92	1.61E-04	0.97				
70	1.09E-04	0.93	1.02E-04	0.91				

C)

	$g_\perp$ (I = 3/2)		$g_\perp$ (I = 0)		$g_{\parallel}$ (I = 0)	
T	T_1	beta	T_1	beta	T_1	beta
15	1.85E-02	0.61	1.04E-02	0.58	1.91E-02	0.61
40	8.32E-04	0.88	7.28E-04	0.93	1.31E-03	0.88
60	2.26E-04	0.92	2.19E-04	0.93	3.73E-04	0.89
70	1.29E-04	0.94	1.33E-04	0.94	2.35E-04	0.88

Table S3. Experimental inversion recovery data for CrN(pyrdtc)$_2$.
In doped Re solid at X-band (**A**); in CH_2Cl_2:toluene at X-band (**B**); in doped Re solid at Q-band (**C**). The experimental inversion recovery data were fitted with a stretched exponential, Eq. 15 in the main text.

A)

Adir (S^{-1} K^{-1})	Aloc (s^{-1})	del (cm^{-1})	Aloc2 (s^{-1})	del2 (cm^{-1})	test = residuals
0.55	$0.55x10^4$	55	$2.0x10^5$	200	0.030
	ARam (s^{-1})	theta (cm^{-1})			
0.55	$0.7x10^5$	70	$1.7x10^5$	200	0.030

B)

Adir (S^{-1} K^{-1})	Aloc (s^{-1})	del (cm^{-1})	Aloc2 (s^{-1})	del2 (cm^{-1})	test = residuals
4.0	$1.2x10^4$	42	$2.3x10^5$	145	0.004
	ARam (s^{-1})	theta (cm^{-1})			
4.5	$1.5x10^5$	55	$1.7x10^5$	150	0.005

Table S4. Fitting parameters employed in the local-mode model.
Parameters for $CrN(pyrdtc)_2$ doped in $ReN(pyrdtc)_2$ (**A**); Parameters for $CrN(trop)_2$ in CH_2Cl_2-toluene (**B**).

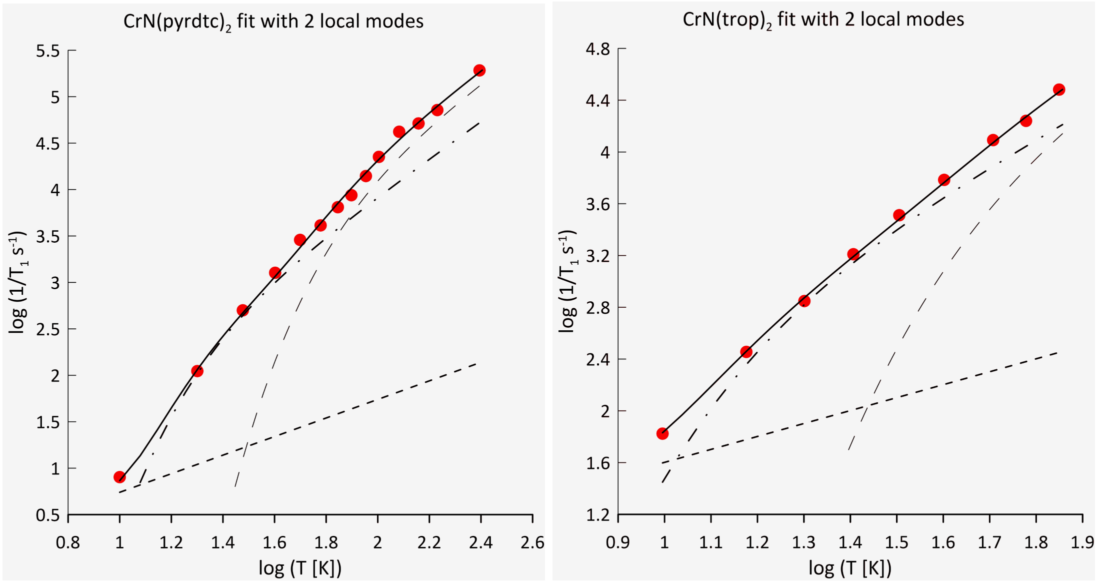

Fig. S3. Local-mode model fitting.

In both plots the solid line is the sum of all contributions, the line with the short dashes has the temperature dependence that is characteristic of the direct process or of spectral diffusion, the dash-dot line is the lower energy local mode, and the line with long dashes in the higher energy local mode.

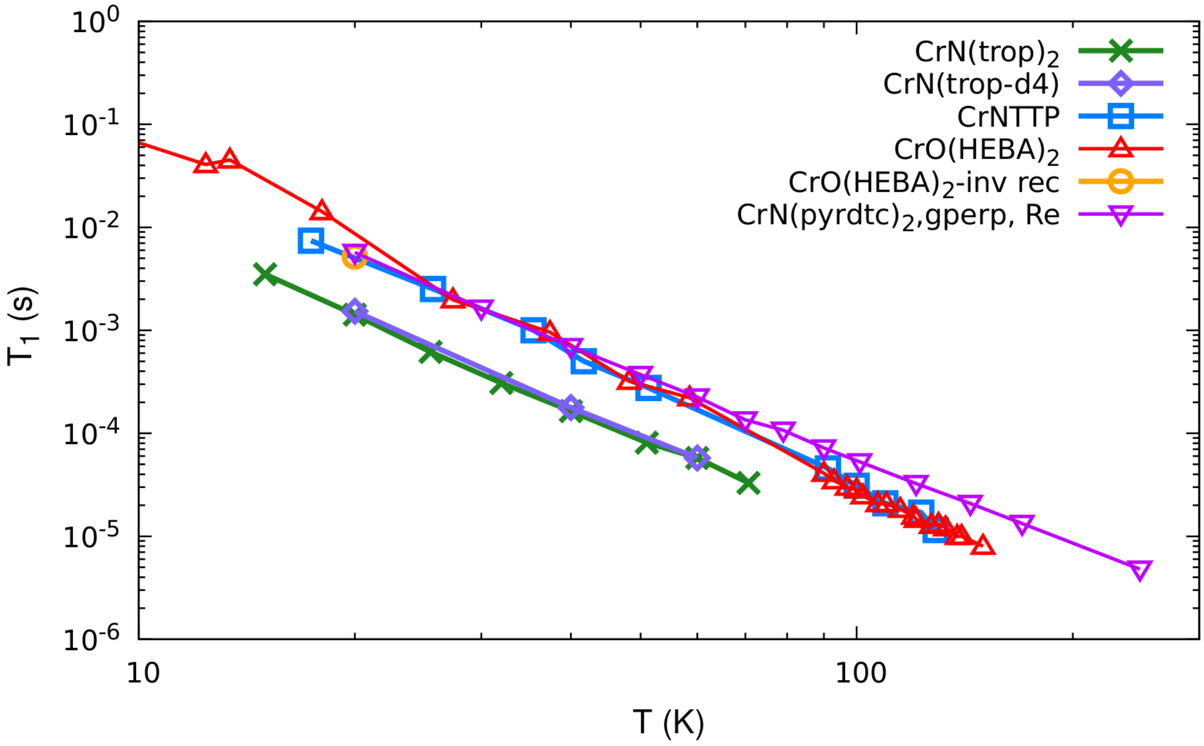

Fig. S4. Comparison of T_1 for $CrN(pyrdtc)_2$ and $CrN(trop)_2$ with previously reported data for other Cr(V) complexes.

The plot includes T_1 data for CrNTTP (chromium(V) nitrido tetraphenyl porphyrin) in toluene solution recorded by saturation recovery as reported in [55], and for $CrO(HEBA)_2$ (chromium (V) bis(2-hydroxyethylbutyrate), in 1:1 water:glycerol recorded by saturation recovery as reported in [17] or by inversion recovery.

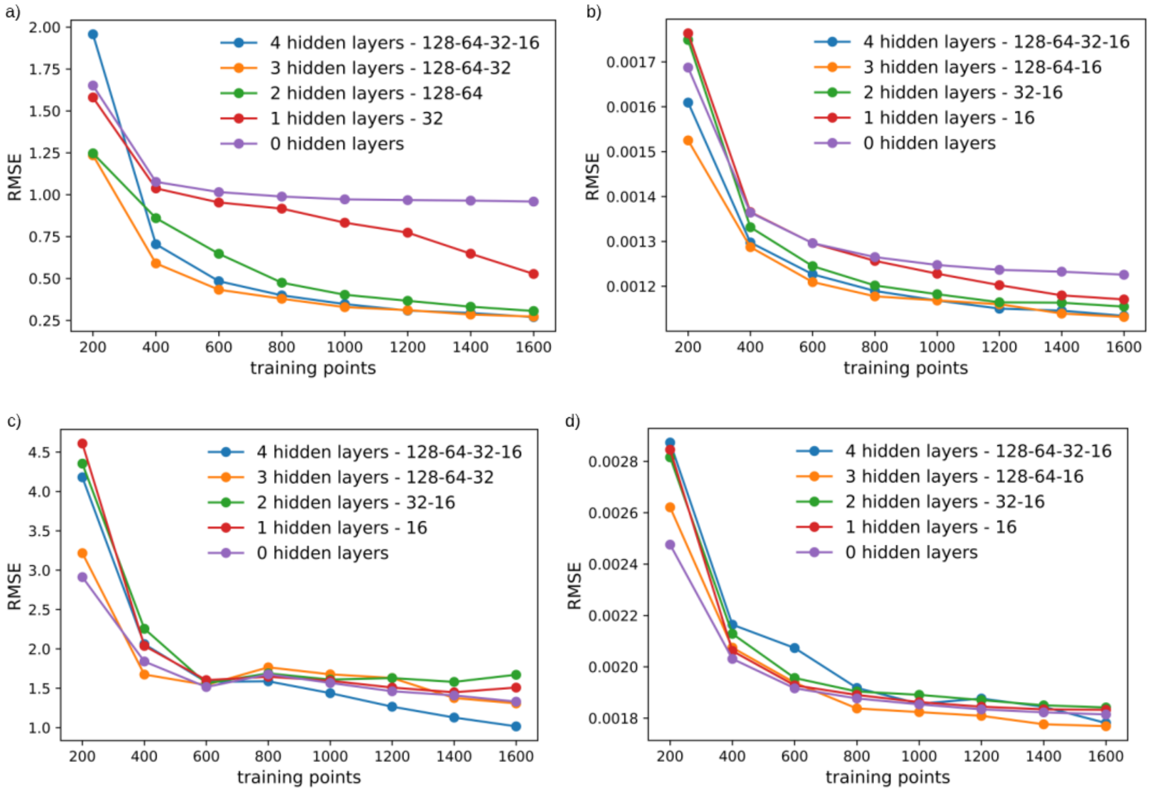

Fig. S5. Comparison of neural network architecture for learning the spin Hamiltonian. The root-mean-squared error (RMSE) of each architecture trained on the **A** tensor of CrN(pyrdtc)$_2$ (**a**); the g tensor of CrN(pyrdtc)$_2$ (**b**); the **A** tensor of CrN(trop)$_2$ (**c**); the g tensor of CrN(trop)$_2$ (**d**). All of the models take Cartesian coordinates as inputs and output the **A**/**g** tensor.

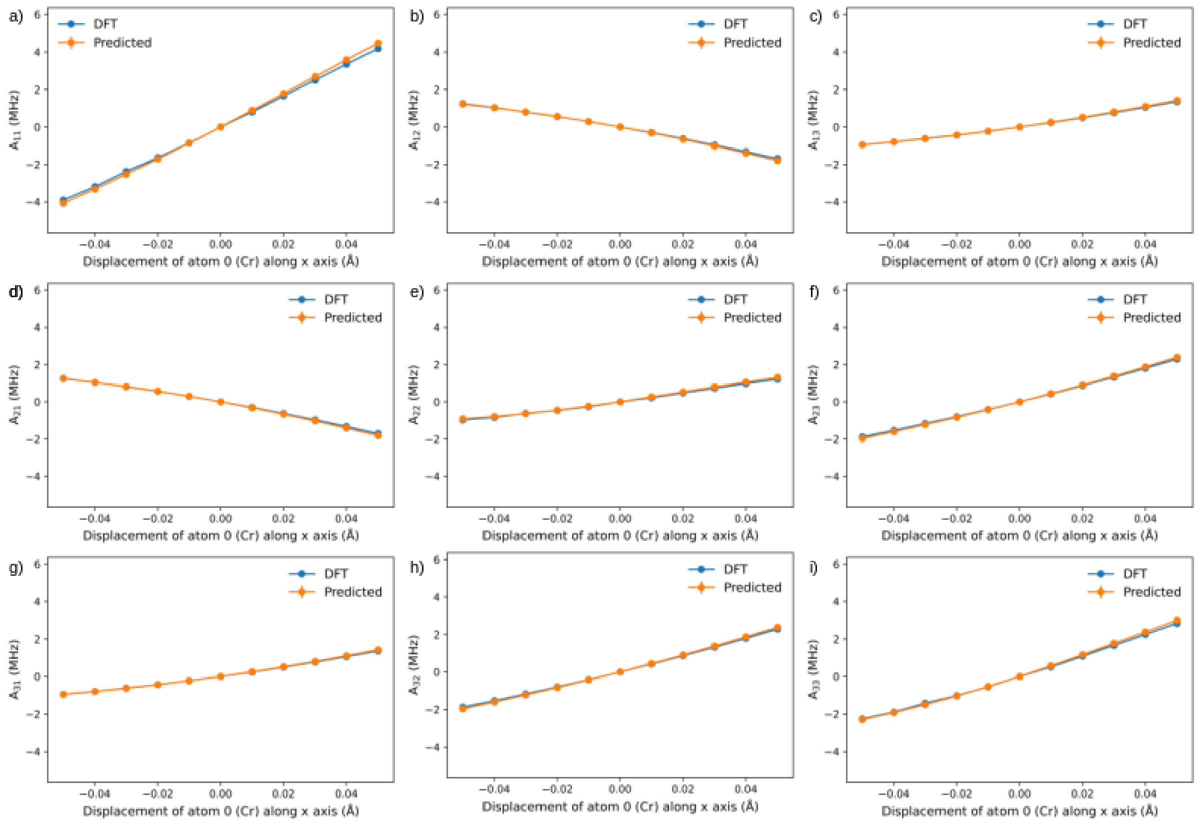

Fig. S6. Comparison of first-order derivatives of the A tensor of CrN(pyrdtc)$_2$. Each component of the **A** tensors (A_{11} (**a**); A_{12} (**b**); A_{13} (**c**); A_{21} (**d**); A_{22} (**e**); A_{23} (**f**); A_{31} (**g**); A_{32} (**h**); A_{33} (**i**)) of CrN(pyrdtc)$_2$ are computed using DFT (blue) and machine learning (orange) when displacing the Cr atom in CrN(pyrdtc)$_2$ along the x direction.

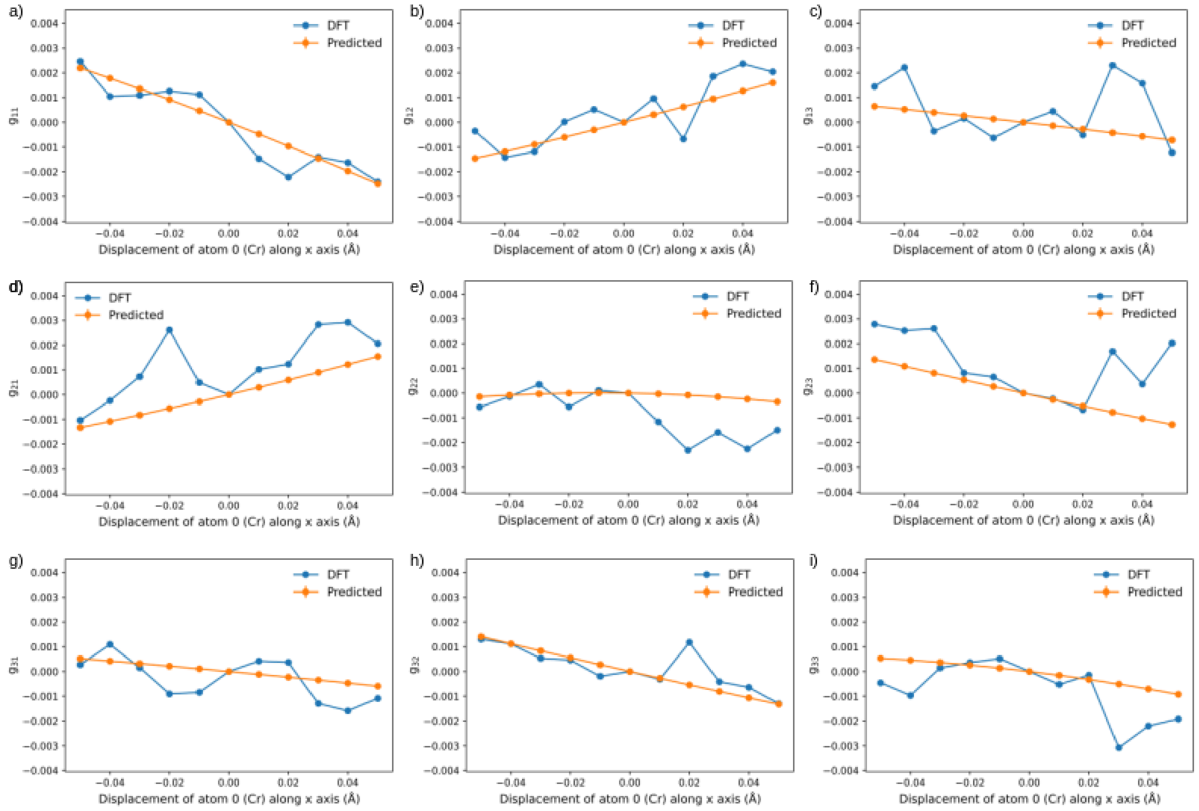

Fig. S7. Comparison of first-order derivatives of the g tensor of CrN(pyrdtc)$_2$.
Each component of the **g** tensors (g_{11} (**a**); g_{12} (**b**); g_{13} (**c**); g_{21} (**d**); g_{22} (**e**); g_{23} (**f**); g_{31} (**g**); g_{32} (**h**); g_{33} (**i**)) of CrN(pyrdtc)$_2$ are computed using DFT (blue) and machine learning (orange) when displacing the Cr atom in CrN(pyrdtc)$_2$ along the x direction.

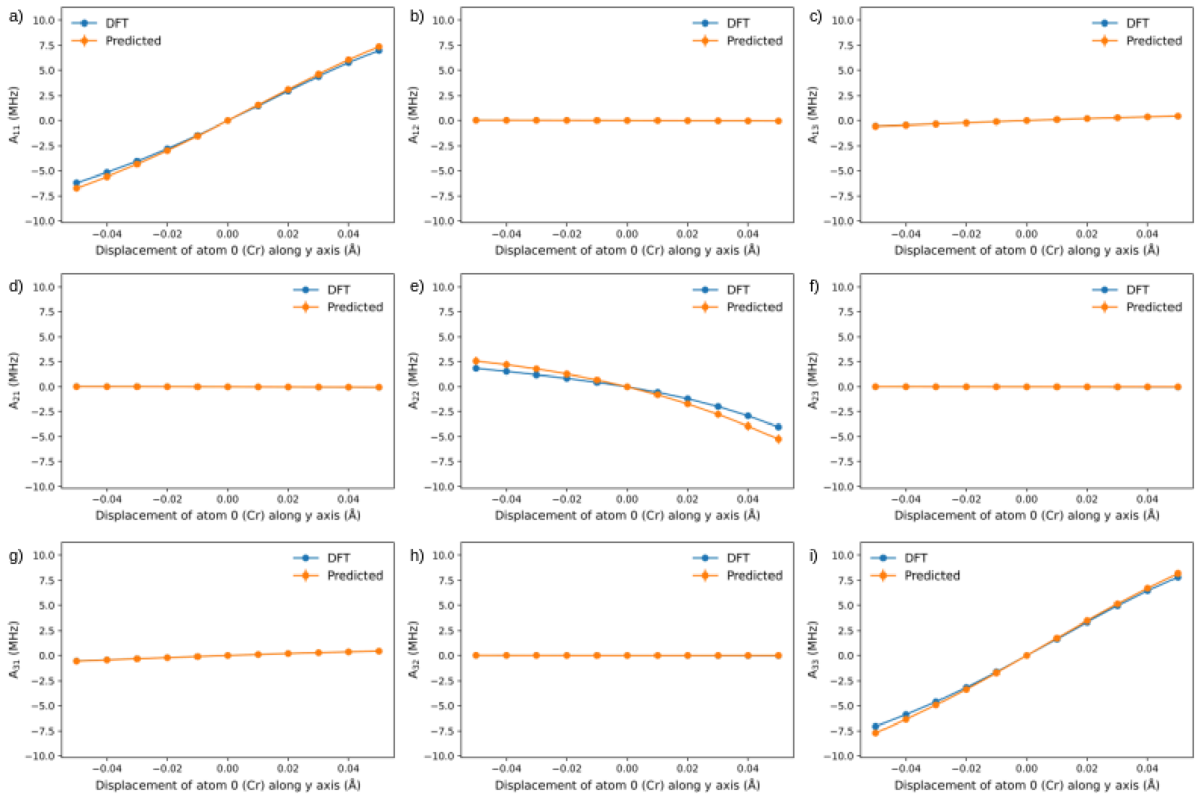

Fig. S8. Comparison of first-order derivatives of the A tensor of CrN(trop)$_2$.
Each component of the A tensors (A_{11} (**a**); A_{12} (**b**); A_{13} (**c**); A_{21} (**d**); A_{22} (**e**); A_{23} (**f**); A_{31} (**g**); A_{32} (**h**); A_{33} (**i**)) of CrN(trop)$_2$ are computed using DFT (blue) and machine learning (orange) when displacing the Cr atom in CrN(trop)$_2$ along the y direction.

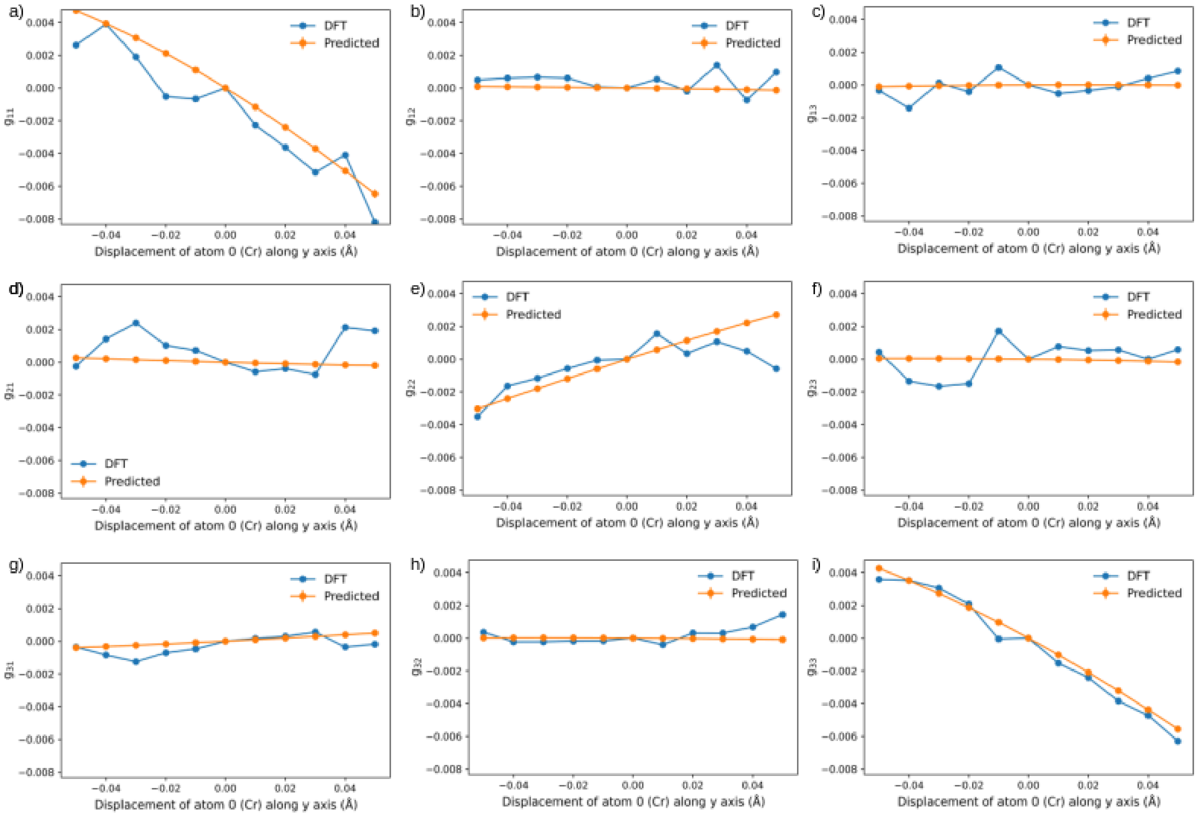

Fig. S9. Comparison of first-order derivatives of the g tensor CrN(trop)$_2$. Each component of the **g** tensors (g_{11} (**a**); g_{12} (**b**); g_{13} (**c**); g_{21} (**d**); g_{22} (**e**); g_{23} (**f**); g_{31} (**g**); g_{32} (**h**); g_{33} (**i**)) of CrN(trop)$_2$ are computed using DFT (blue) and machine learning (orange) when displacing the Cr atom in CrN(trop)$_2$ along the y direction.

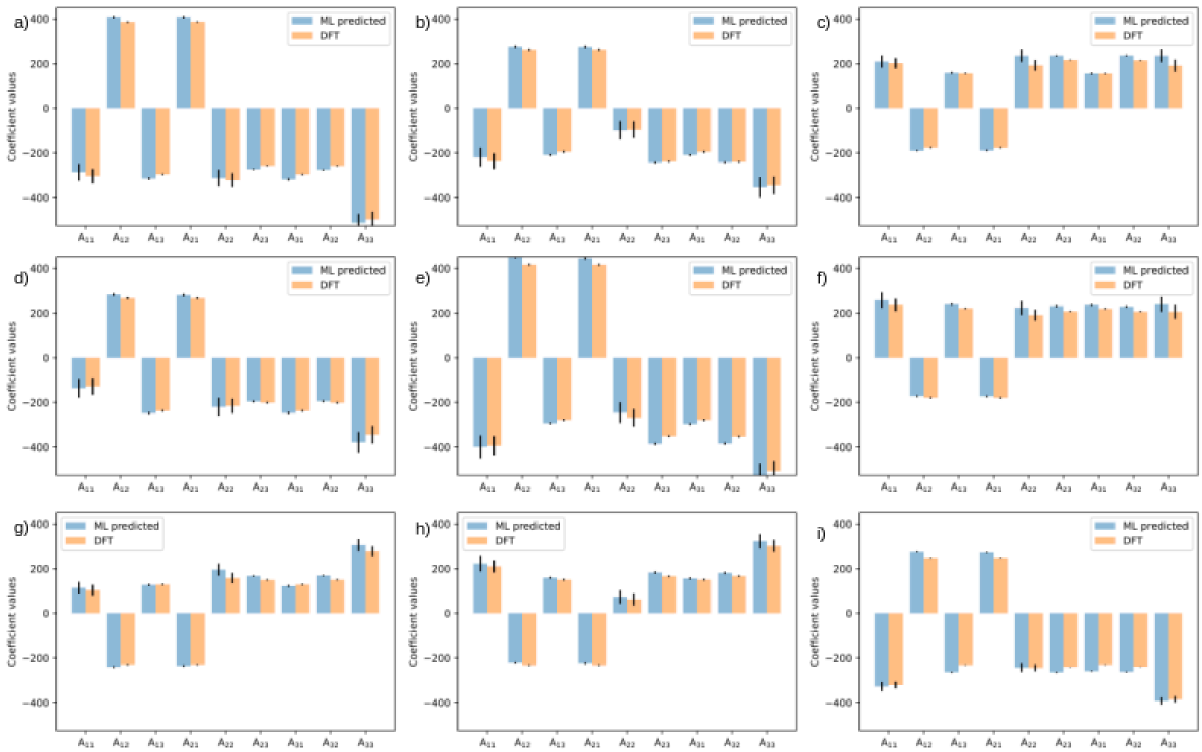

Fig. S10. Comparison of the second-order derivatives of the A tensor of CrN(pyrdtc)$_2$. The second-order derivatives are calculated from each component of the **A** tensors of CrN(pyrdtc)$_2$ computed using DFT (blue) and machine learning (orange) when displacing the Cr and N atoms along the x and x direction (**a**); along the x and y direction (**b**); along the x and z direction (**c**); along the y and x direction (**d**); along the y and y direction (**e**); along the y and z direction (**f**); along the z and x direction (**g**); along the z and y direction (**h**); along the z and z direction (**i**), respectively.

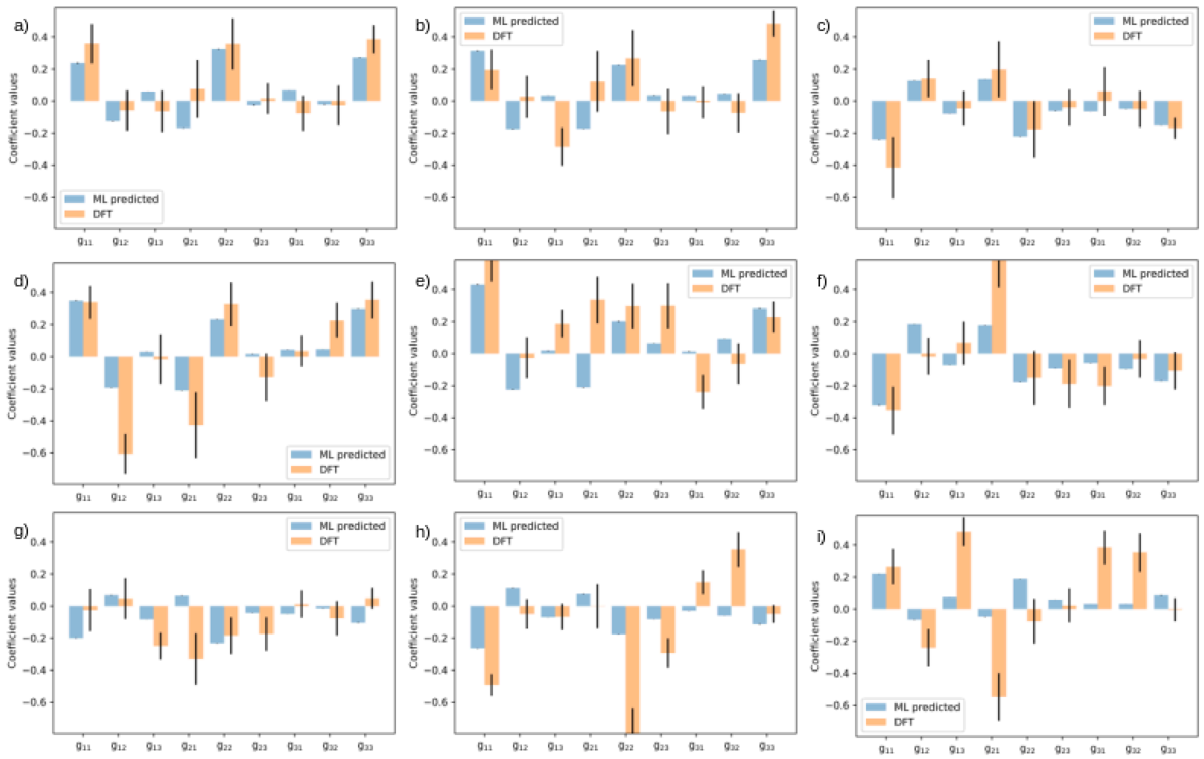

Fig. S11. Comparison of the second-order derivatives of the g tensor of CrN(pyrdtc)$_2$. The second-order derivatives are calculated from each component of the **g** tensors of CrN(pyrdtc)$_2$ computed using DFT (orange) and machine learning (blue) when displacing the Cr and N atoms along the x and x direction (**a**); along the x and y direction (**b**); along the x and z direction (**c**); along the y and x direction (**d**); along the y and y direction (**e**); along the y and z direction (**f**); along the z and x direction (**g**); along the z and y direction (**h**); along the z and z direction (**i**), respectively.

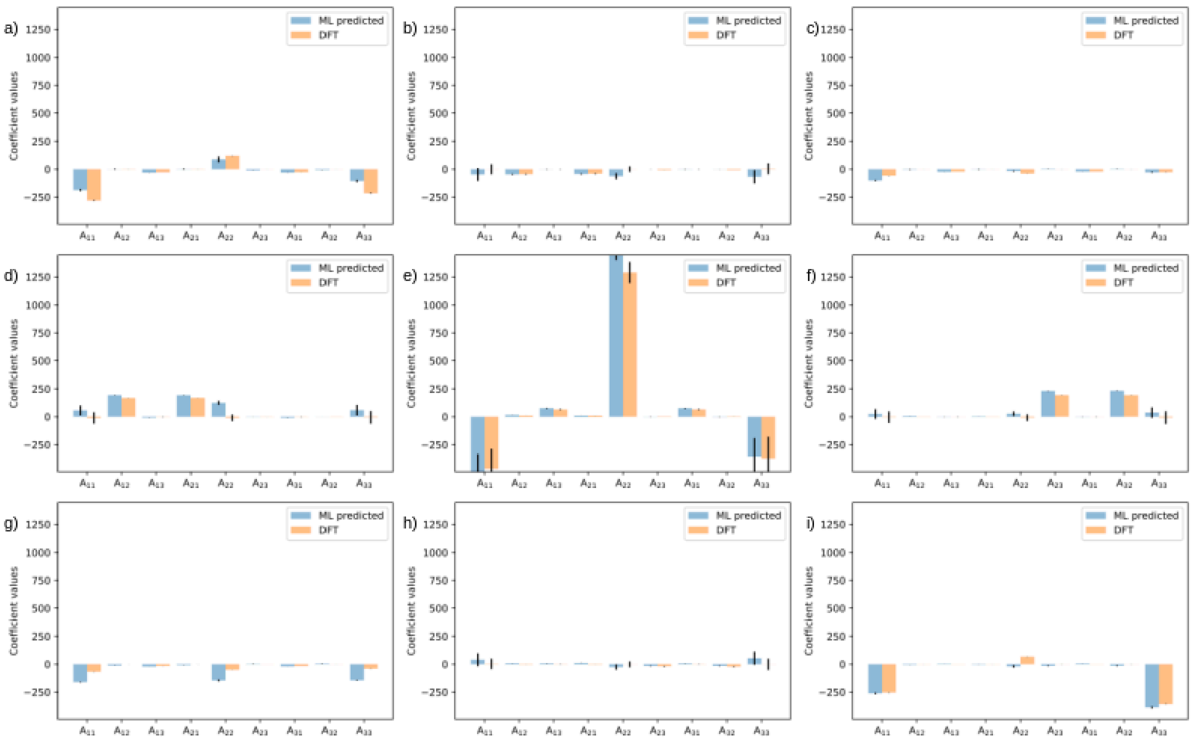

Fig. S12. Comparison of the second-order derivatives of the A tensor of CrN(trop)$_2$. The second-order derivatives are calculated from each component of the **A** tensors of CrN(trop)$_2$ computed using DFT (blue) and machine learning (orange) when displacing the Cr and N atoms along the x and x direction (**a**); along the x and y direction (**b**); along the x and z direction (**c**); along the y and x direction (**d**); along the y and y direction (**e**); along the y and z direction (**f**); along the z and x direction (**g**); along the z and y direction (**h**); along the z and z direction (**i**), respectively.

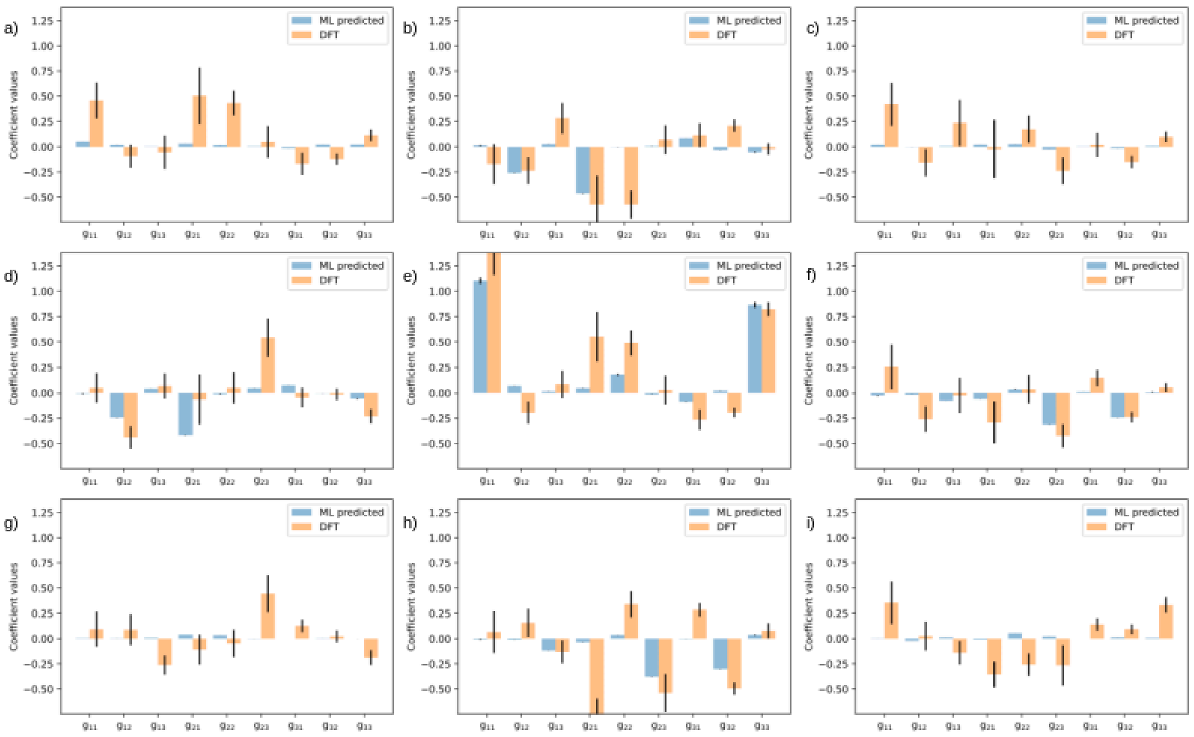

Fig. S 13. Comparison of the second-order derivatives of the g tensor of $CrN(trop)_2$. The second-order derivatives are calculated from each component of the **g** tensors of $CrN(trop)_2$ computed using DFT (blue) and machine learning (orange) when displacing the Cr and N atoms along the x and x direction (**a**); along the x and y direction (**b**); along the x and z direction (**c**); along the y and x direction (**d**); along the y and y direction (**e**); along the y and z direction (**f**); along the z and x direction (**g**); along the z and y direction (**h**); along the z and z direction (**i**), respectively.

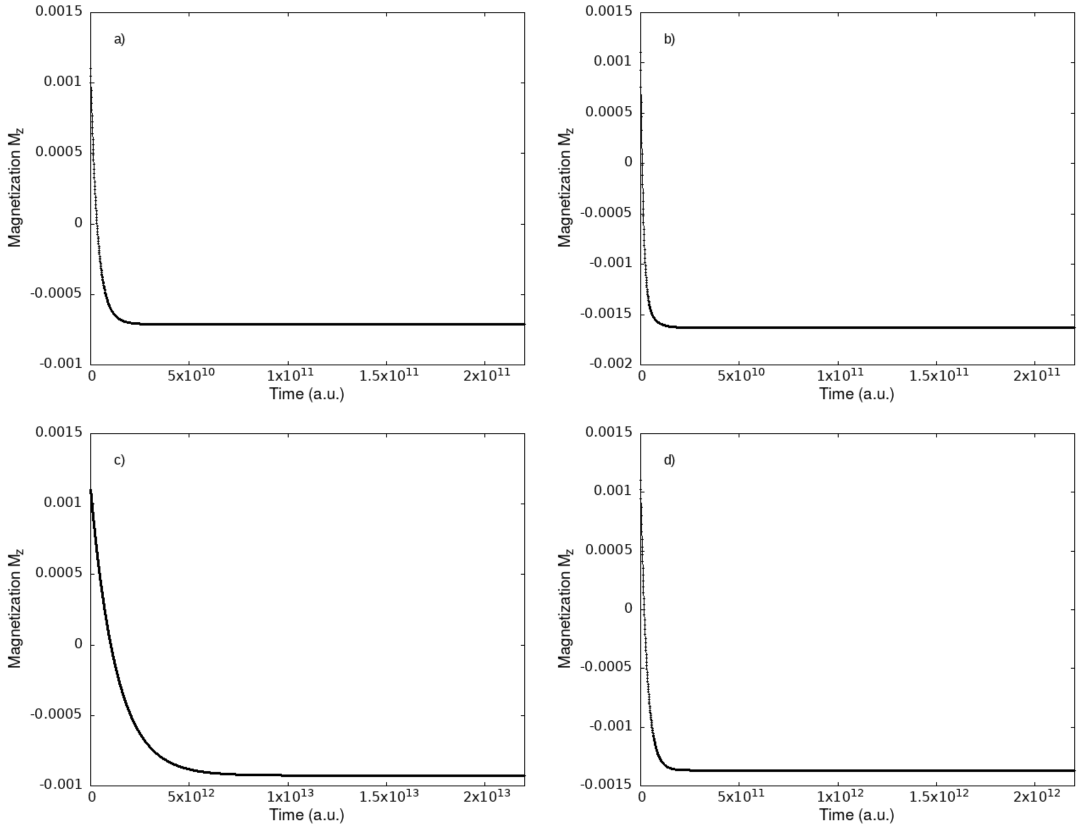

Fig. S14. Magnetization decay.

Computed $M_z(t)$ from **A** tensor for $CrN(pyrdtc)_2$ (**a**) and CrN(trop)2 (**b**); from **g** tensor for $CrN(pyrdtc)_2$ (**c**) and CrN(trop)2 (**d**). The reported values are computed for X-Band and with the molecule parallel to the applied magnetic field.

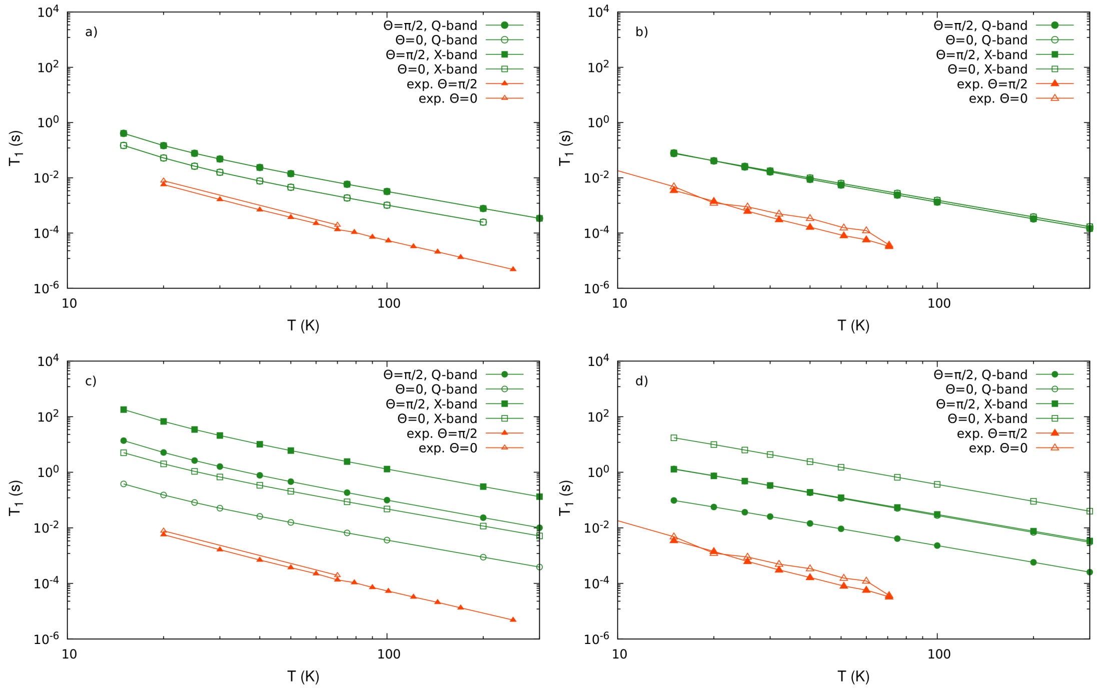

Fig. S15. Γ_I relaxation mechanism's angular dependency.
Temperature dependence of spin-lattice relaxation time T_1 computed considering Γ_I mechanism (Eq. 8 in the main text). In each plot, the orientation of the molecule relative to the applied magnetic field, as well as the intensity of the magnetic field, is reported. Relaxation from **A** tensor for $CrN(pyrdtc)_2$ (**a**) and CrN(trop)2 (**b**); Relaxation from **g** tensor for $CrN(pyrdtc)_2$ (**c**) and $CrN(trop)_2$ (**d**).

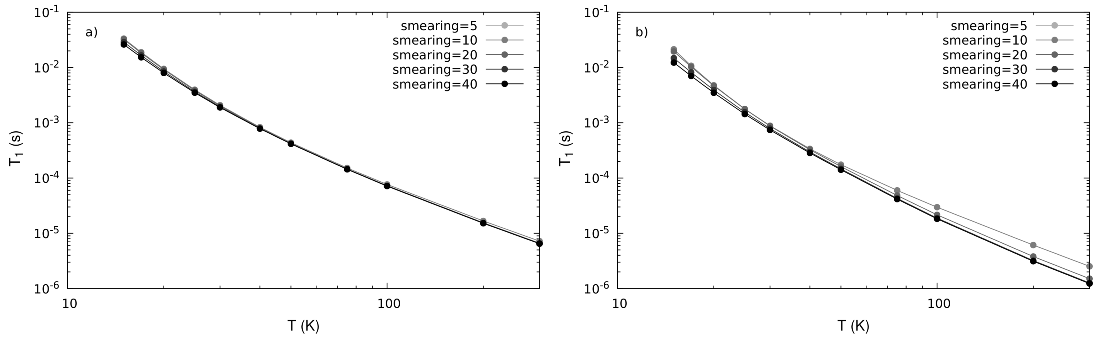

Fig. S16. Γ_{II} relaxation mechanism's dependency on Gaussian smearing.

T_1 relaxation time computed for different values of gaussian smearing (cm^{-1}) for CrN(pyrdtc)$_2$ (**a**) and CrN(trop)2 (**b**).

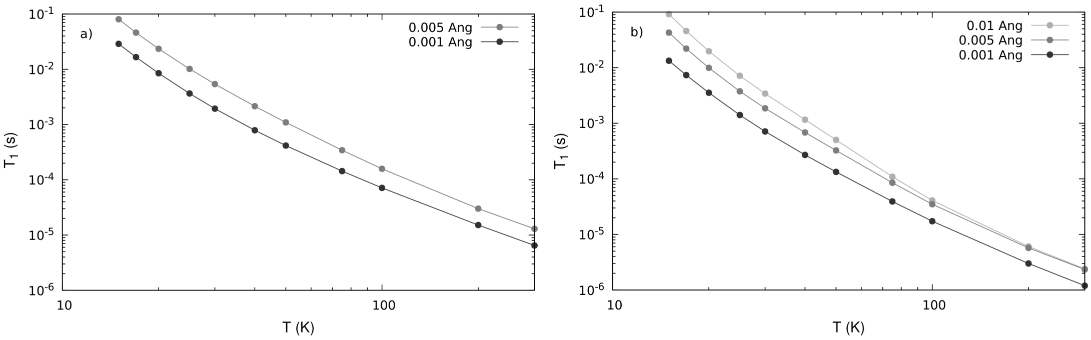

Fig. S17. Γ_{II} relaxation mechanism's dependency on numerical differentiation step. T_1 relaxation time computed for different values of geometrical displacement employed for the evaluation of the non-adiabatic coupling vectors in Eq. 16 in the main text. CrN(pyrdtc)$_2$ (**a**) and CrN(trop)$_2$ (**b**).

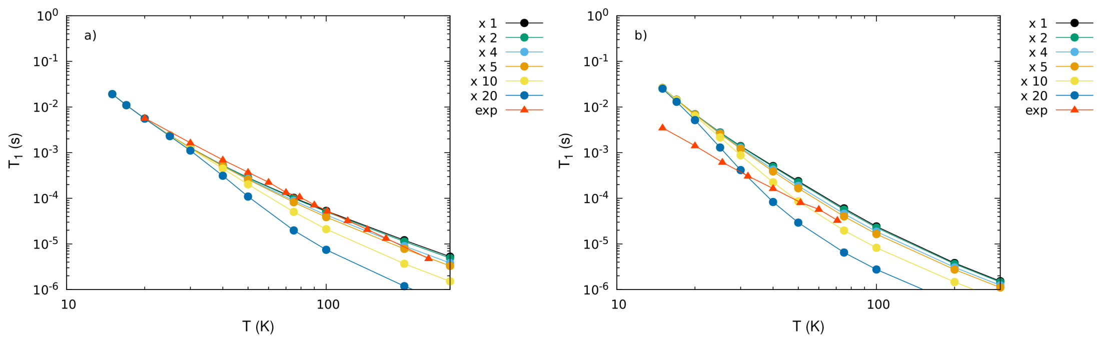

Fig. S18. Γ_{II} relaxation mechanism's dependency on phonons from local-mode model. T_1 relaxation time computed by multiplying the terms in Eq. 12 (main text) relative to high-energy local modes (del2 of Table S4) by different factors (1, 2, 4, 5, 10, 20). CrN(pyrdtc)$_2$ with augmented rate population transfer for phonons energy between 180 and 220 cm^{-1} (**a**). CrN(trop)$_2$ with augmented rate population transfer for phonons energy between 130 and 170 cm^{-1} (**b**).

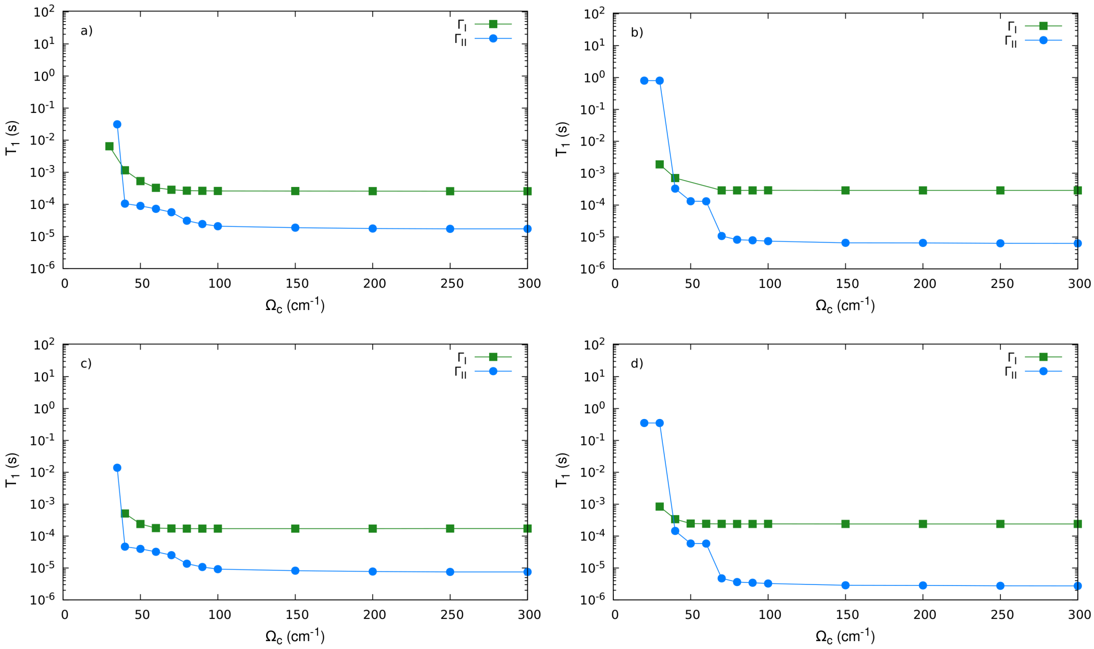

Fig. S19. Γ_I and Γ_{II} relaxation with high-energy phonon cutoff for different temperatures.

T_1 relaxation time computed as for in Figure 4A in the main text. CrN(pyrdtc)$_2$ at 200K (**a**). CrN(trop)$_2$ at 200K (**b**). CrN(pyrdtc)$_2$ at 300K (**c**). CrN(trop)$_2$ at 300K (**d**).

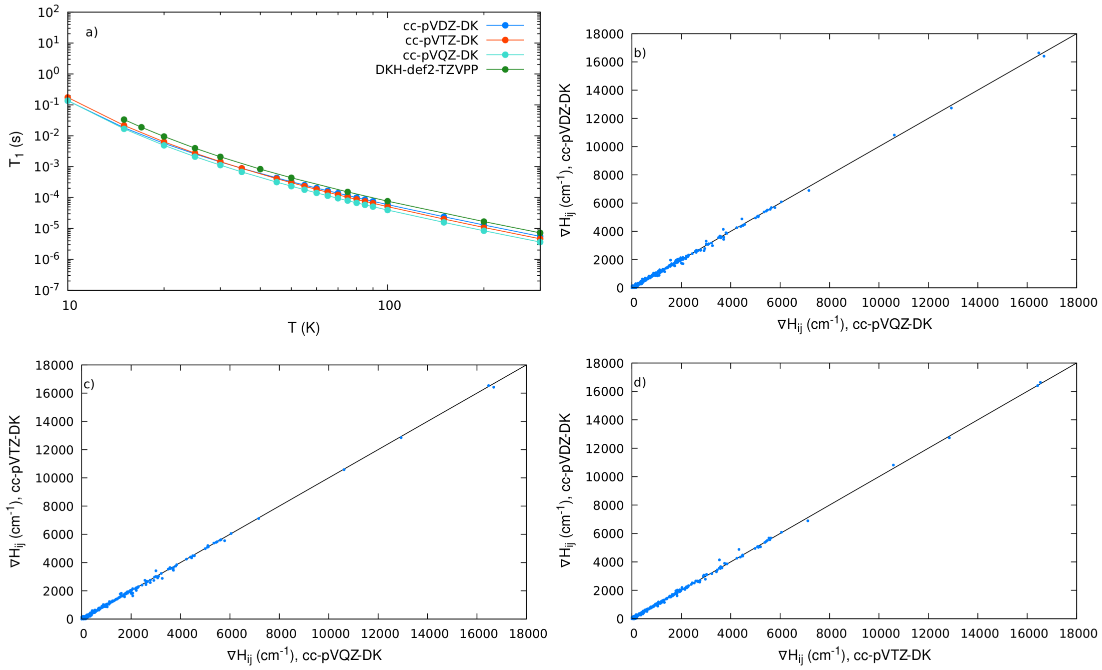

Fig. S20. CrN(pyrdtc)$_2$ basis set dependency at NEV(1,5) level of theory.
T_1 relaxation time computed using different basis sets (**a**). Comparison of ∇H_{ij} matrix elements (in cm^{-1}) between cc-pVQZ-DK and cc-pVDZ-DK basis sets (**b**). Comparison of ∇H_{ij} matrix elements (in cm^{-1}) betweem cc-pVQZ-DK and cc-pVTZ-DK basis sets (**c**). Comparison of ∇H_{ij} matrix elements (in cm^{-1}) between cc-pVTZ-DK and cc-pVDZ-DK basis sets (**d**).

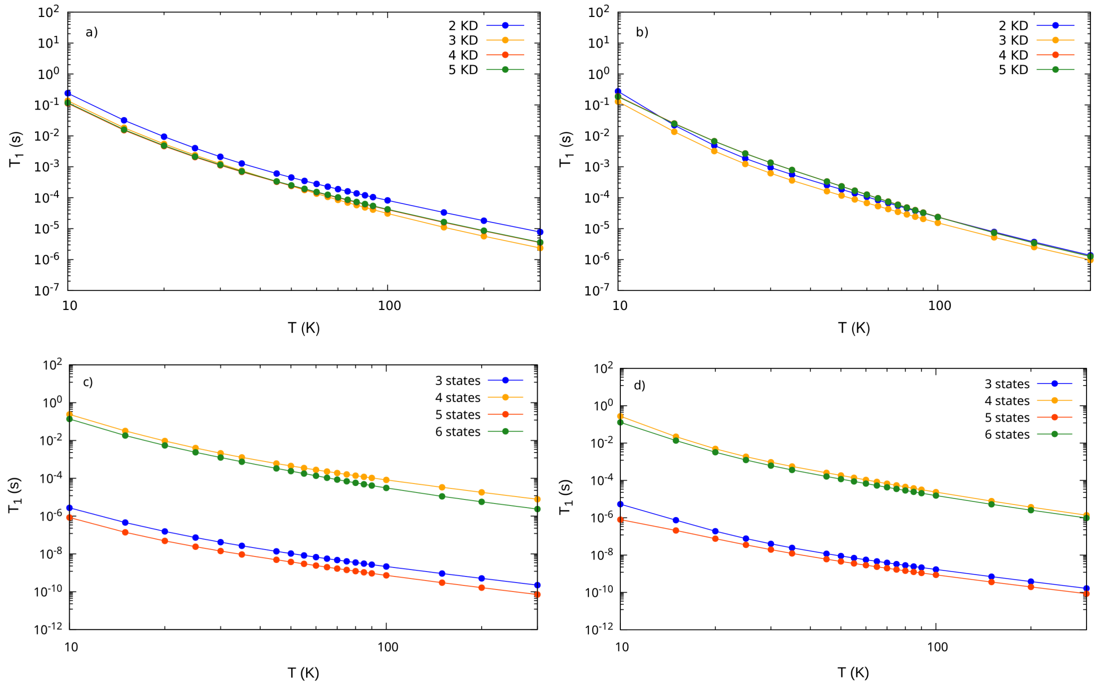

Fig. S21. Γ_{II} relaxation mechanism's dependency on number of Kramers pairs and electronic ab initio states included in the calculation

T_1 relaxation time computed including different numbers of Kramers Doublets (KD) in the evaluation of Eq. 11 of the main text for CrN(pyrdtc)$_2$ (**a**) and CrN(trop)$_2$ (**b**). T_1 relaxation time computed including different numbers of electronic ab initio states in the evaluation of Eq. 11 of the main text for CrN(pyrdtc)$_2$ (**c**) and CrN(trop)$_2$ (**d**).

5 KDs (i.e. 10 electronic ab initio states) is the number of Kramers pairs considered for the simulations in Fig. 3 of the main text.

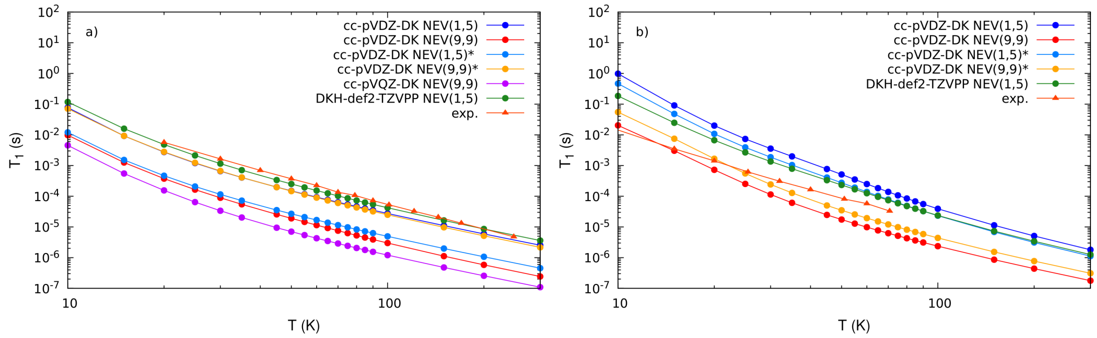

Fig. S22. Γ_{II} relaxation mechanism's dependency on the level of theory.
T_1 relaxation time computed for different methods and basis sets in comparison with experimental data. For the methods marked with '*', the vibronic coupling matrix elements are obtained with the reported method, but the static energy contribution is extracted from NEV(1,5) in the case of NEV(9,9)* (orange dots), while static energy contribution is extracted from NEV(9,9) in the case of NEV(1,5)* (light blue dots). $CrN(pyrdtc)_2$ (**a**). $CrN(trop)_2$ (**b**).

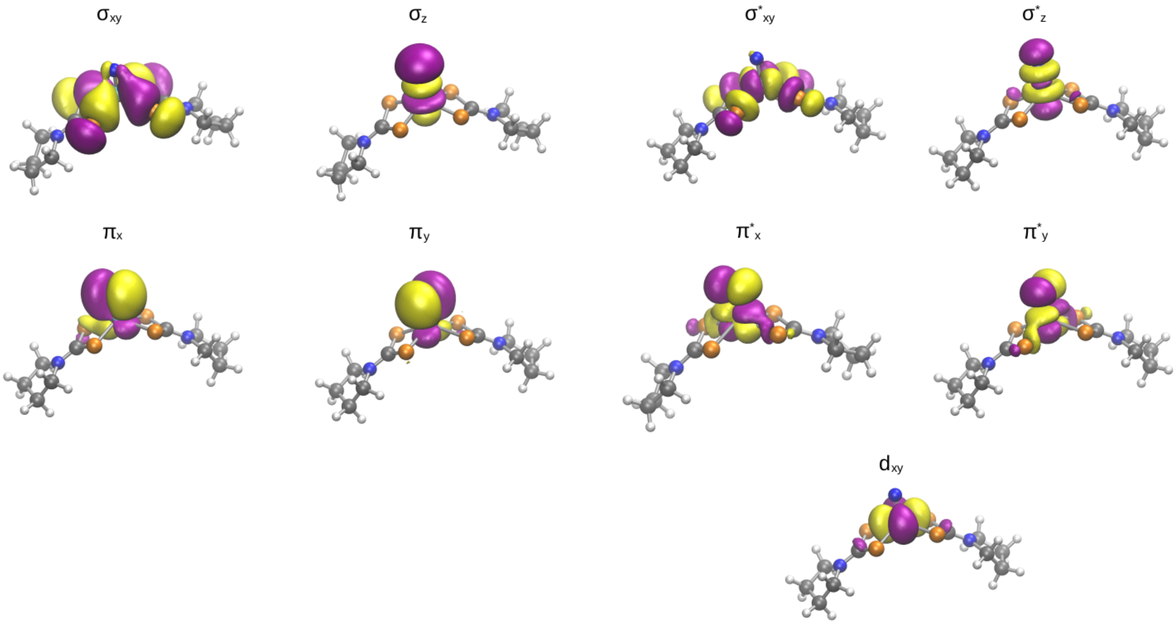

Fig. S23. CrN(pyrdtc)$_2$ CAS(9,9) active orbitals.
CAS(9,9) active orbitals plotted using an isovalue of 0.02 e/Å^3. For the calculation, the DKH-def2-TZVPP basis set was employed.

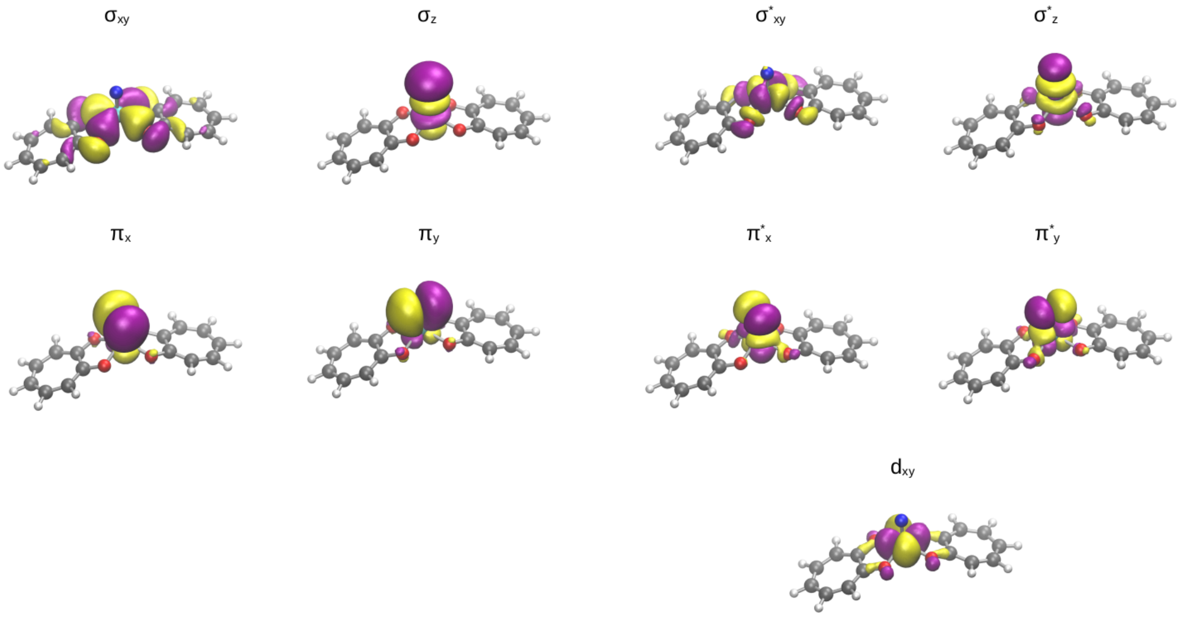

Fig. S24. CrN(trop)$_2$ CAS(9,9) active orbitals.
CAS(9,9) active orbitals plotted using an isovalue of 0.02 e/Å^3. For the calculation, the DKH-def2-TZVPP basis set was employed.

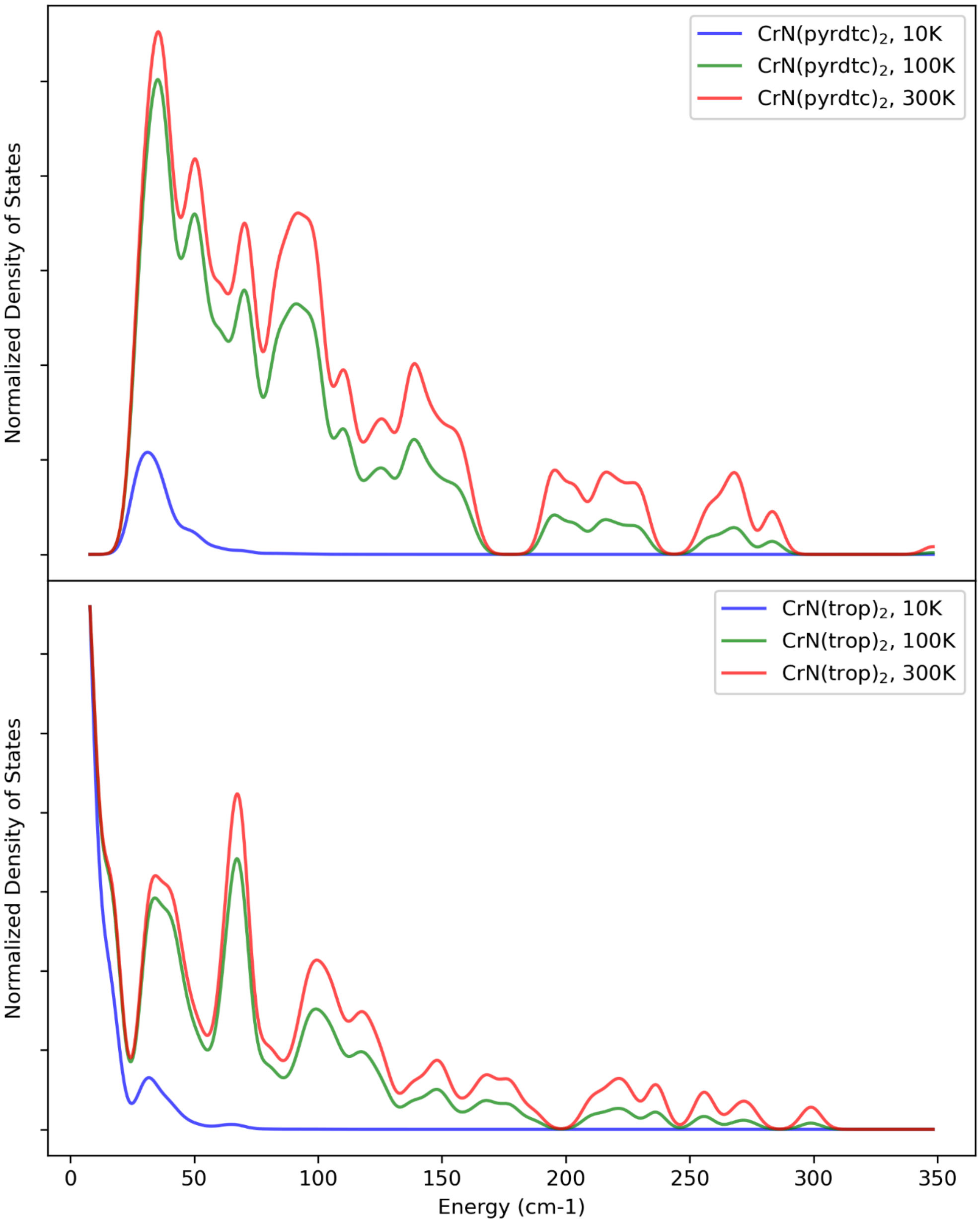

CrN(pyrdtc)$_2$, 10K
CrN(pyrdtc)$_2$, 100K
CrN(pyrdtc)$_2$, 300K
Normalized Density of States
CrN(trop)$_2$, 10K
CrN(trop)$_2$, 100K
CrN(trop)$_2$, 300K
Normalized Density of States
0
50
100
150
200
250
300
350
Energy (cm-1)

Fig. S25. Weighted phonon spectra.
Normalized phonon density of states weighted by the Bose-Einstein factor at different temperatures: 10 K (blue), 100 K (green), and 300 K (red).

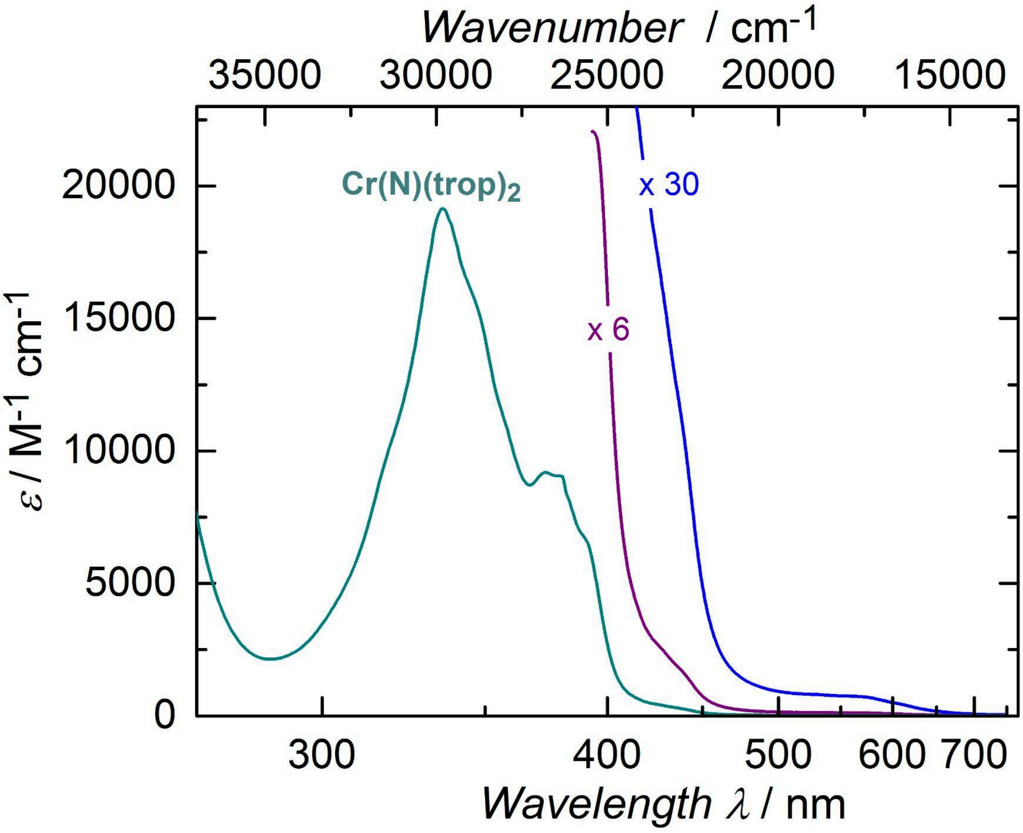

Fig. S26. US-VIS spectra for CrN(trop)$_2$.
The spectra was obtained for a CH_2Cl_2 solution with a Perkin Elmer Lambda 2 spectrometer.

	Transition	CASSCF	NEVPT2	Experiment
KD 1	d_{xy} (99%)	0	0	
KD 2	$d_{xy} \rightarrow d_{x2-y2}$ (95%)	13887.0	29680.4	18248
KD 3	$d_{xy} \rightarrow d_{xz}$ (97%)	22622.1	45734.3	22422
KD 4	$d_{xy} \rightarrow d_{yz}$ (93%)	24498.5	47961.0	23148
KD 5	$d_{xy} \rightarrow d_{z2}$ (99%)	32830.9	54774.5	36232

Table S5. $CrN(pyrdtc)_2$ excited states for active space (1,5) and DKH-def2-TZVPP basis set.

Excitations from the ground state Kramers Doublet (KD) in cm^{-1}. Values computed with both CASSCF and NEVPT2 methods are reported and compared with experimental results taken from ref. 51. The character and weight of the main component of each CAS wavefunction is reported in the second column.

	Transition	CASSCF	NEVPT2	Experiment
KD 1	d_{xy} (73 %)	0	0	
KD 2	$d_{xy} \rightarrow \sigma^*_{xy}$ (72 %)	17550.4	20323.4	18248
KD 3	$\pi_y \rightarrow d_{xy}$ (54 %)	31822.8	28182.7	22422
KD 4	$\pi_x \rightarrow d_{xy}$ (58 %)	32002.3	28430.6	23148
KD 5	$\pi_x \rightarrow \sigma^*_{xy}$ (53 %)	36929.5	36124.8	36232

Table S6. CrN(pyrdtc)$_2$ excited states for active space (9,9) and DKH-def2-TZVPP basis set.

Excitations from the ground state Kramers Doublet (KD) in cm^{-1} . Values computed with both CASSCF and NEVPT2 methods are reported and compared with experimental results taken from ref. 51. The character and weight of the main component of each CAS wavefunction is reported in the second column.

	Transition	CASSCF	NEVPT2	Experiment
KD 1	d_{xy} (99%)	0	0	
KD 2	$d_{xy} \rightarrow d_{x2-y2}$ (92%)	13894.1	29663.9	18248
KD 3	$d_{xy} \rightarrow d_{xz}$ (98%)	22626.2	45577.1	22422
KD 4	$d_{xy} \rightarrow d_{yz}$ (90%)	24741.4	48253.7	23148
KD 5	$d_{xy} \rightarrow d_{z2}$ (99%)	32827.7	54947.4	36232

Table S7. CrN(pyrdtc)$_2$ excited states for active space (1,5) and cc-pVDZ-DK basis set. Excitations from the ground state Kramers Doublet (KD) in cm^{-1} . Values computed with both CASSCF and NEVPT2 methods are reported and compared with experimental results taken from ref. 51. The character and weight of the main component of each CAS wavefunction is reported in the second column.

	Transition	CASSCF	NEVPT2	Experiment
KD 1	d_{xy} (73 %)	0	0	
KD 2	$d_{xy} \rightarrow \sigma^*_{xy}$ (72 %)	17581.6	20585.5	18248
KD 3	$\pi_y \rightarrow d_{xy}$ (39 %)	31894.0	28605.1	22422
KD 4	$\pi_x \rightarrow d_{xy}$ (43 %)	32122.8	28874.4	23148
KD 5	$\pi_x \rightarrow \sigma^*_{xy}$ (53 %)	36996.4	36393.2	36232

Table S8. CrN(pyrdtc)$_2$ excited states for active space (9,9) and cc-pVDZ-DK basis set. Excitations from the ground state Kramers Doublet (KD) in cm^{-1} . Values computed with both CASSCF and NEVPT2 methods are reported and compared with experimental results taken from ref. 51. The character and weight of the main component of each CAS wavefunction is reported in the second column.

	Transition	CASSCF	NEVPT2	Experiment
KD 1	d_{xy} (99%)	0	0	
KD 2	$d_{xy} \rightarrow d_{x2-y2}$ (95%)	13862.4	29601.7	18248
KD 3	$d_{xy} \rightarrow d_{xz}$ (97%)	22603.0	45691.9	22422
KD 4	$d_{xy} \rightarrow d_{yz}$ (92%)	24521.4	48008.8	23148
KD 5	$d_{xy} \rightarrow d_{z2}$ (99%)	32859.0	54736.3	36232

Table S9. CrN(pyrdtc)$_2$ excited states for active space (1,5) and cc-pVTZ-DK basis set. Excitations from the ground state Kramers Doublet (KD) in cm^{-1} . Values computed with both CASSCF and NEVPT2 methods are reported and compared with experimental results taken from ref. 51. The character and weight of the main component of each CAS wavefunction is reported in the second column.

	Transition	CASSCF	NEVPT2	Experiment
KD 1	d_{xy} (73 %)	0	0	
KD 2	$d_{xy} \rightarrow \sigma^*_{xy}$ (72 %)	17524.7	20307.2	18248
KD 3	$\pi_y \rightarrow d_{xy}$ (51 %)	31827.8	28194.1	22422
KD 4	$\pi_x \rightarrow d_{xy}$ (55 %)	32005.9	28446.1	23148
KD 5	$\pi_x \rightarrow \sigma^*_{xy}$ (54 %)	36906.8	36141.5	36232

Table S10. $CrN(pyrdtc)_2$ excited states for active space (9,9) and cc-pVTZ-DK basis set. Excitations from the ground state Kramers Doublet (KD) in cm^{-1} . Values computed with both CASSCF and NEVPT2 methods are reported and compared with experimental results taken from ref. 51. The character and weight of the main component of each CAS wavefunction is reported in the second column.

	Transition	CASSCF	NEVPT2	Experiment
KD 1	d_{xy} (99%)	0	0	
KD 2	$d_{xy} \rightarrow d_{x2-y2}$ (94%)	13849.5	29619.7	18248
KD 3	$d_{xy} \rightarrow d_{xz}$ (97%)	22550.2	45936.8	22422
KD 4	$d_{xy} \rightarrow d_{yz}$ (92%)	24424.9	48100.6	23148
KD 5	$d_{xy} \rightarrow d_{z2}$ (99%)	32845.7	54865.8	36232

Table S11. CrN(pyrdtc)$_2$ excited states for active space (1,5) and cc-pVQZ-DK basis set. Excitations from the ground state Kramers Doublet (KD) in cm^{-1}. Values computed with both CASSCF and NEVPT2 methods are reported and compared with experimental results taken from ref. 51. The character and weight of the main component of each CAS wavefunction is reported in the second column.

	Transition	CASSCF	NEVPT2	Experiment
KD 1	d_{xy} (73 %)	0	0	
KD 2	$d_{xy} \rightarrow \sigma^*_{xy}$ (72 %)	17528.5	20238.3	18248
KD 3	$\pi_y \rightarrow d_{xy}$ (50 %)	31800.1	27915.6	22422
KD 4	$\pi_x \rightarrow d_{xy}$ (55 %)	31967.3	28158.2	23148
KD 5	$\pi_x \rightarrow \sigma^*_{xy}$ (54 %)	36888.2	36005.9	36232

Table S12. $CrN(pyrdtc)_2$ excited states for active space (9,9) and cc-pVQZ-DK basis set. Excitations from the ground state Kramers Doublet (KD) in cm^{-1}. Values computed with both CASSCF and NEVPT2 methods are reported and compared with experimental results taken from ref. 51. The character and weight of the main component of each CAS wavefunction is reported in the second column.

	Transition	CASSCF	NEVPT2	Experiment
KD 1	d_{xy} (99%)	0	0	
KD 2	$d_{xy} \rightarrow d_{x2-y2}$ (97%)	15610.8	21515.8	18000
KD 3	$d_{xy} \rightarrow d_{xz}$ (99%)	21717.4	37694.1	
KD 4	$d_{xy} \rightarrow d_{yz}$ (99%)	25066.2	41994.8	
KD 5	$d_{xy} \rightarrow d_{z2}$ (97%)	35546.4	52834.2	

Table S13. $CrN(trop)_2$ excited states for active space (1,5) and DKH-def2-TZVPP basis set.

Excitations from the ground state Kramers Doublet (KD) in cm^{-1} . Values computed with both CASSCF and NEVPT2 methods are reported and compared with experimental results (Fig. S26). The character and weight of the main component of each CAS wavefunction is reported in the second column.

	Transition	CASSCF	NEVPT2	Experiment
KD 1	d_{xy} (73 %)	0	0	
KD 2	$d_{xy} \rightarrow \sigma^*_{xy}$ (73 %)	17053.9	19156.7	18000
KD 3	$\pi_y \rightarrow d_{xy}$ (50 %)	31569.3	28171.9	
KD 4	$\pi_x \rightarrow d_{xy}$ (57 %)	32242.2	28741.0	
KD 5	$\pi_x \rightarrow \sigma^*_{xy}$ (34 %)	36633.4	34472.5	

Table S14. CrN(trop)$_2$ excited states for active space (9,9) and DKH-def2-TZVPP basis set.
Excitations from the ground state Kramers Doublet (KD) in cm^{-1}. Values computed with both CASSCF and NEVPT2 methods are reported and compared with experimental results (Fig. S26). The character and weight of the main component of each CAS wavefunction is reported in the second column.

	Transition	CASSCF	NEVPT2	Experiment
KD 1	d_{xy} (99%)	0	0	
KD 2	$d_{xy} \rightarrow d_{x2-y2}$ (97%)	15564.2	21499.9	18000
KD 3	$d_{xy} \rightarrow d_{xz}$ (99%)	21768.1	37866.7	
KD 4	$d_{xy} \rightarrow d_{yz}$ (99%)	25233.8	42264.1	
KD 5	$d_{xy} \rightarrow d_{z2}$ (97%)	35558.5	53324.6	

Table S15. $CrN(trop)_2$ excited states for active space (1,5) and cc-pVDZ-DK basis set. Excitations from the ground state Kramers Doublet (KD) in cm^{-1} . Values computed with both CASSCF and NEVPT2 methods are reported and compared with experimental results (Fig. S26). The character and weight of the main component of each CAS wavefunction is reported in the second column.

	Transition	CASSCF	NEVPT2	Experiment
KD 1	d_{xy} (73 %)	0	0	
KD 2	$d_{xy} \rightarrow \sigma^*_{xy}$ (73 %)	17035.8	19282.7	18000
KD 3	$\pi_y \rightarrow d_{xy}$ (51 %)	31599.9	28557.9	
KD 4	$\pi_x \rightarrow d_{xy}$ (57 %)	32286.0	29115.0	
KD 5	$\pi_x \rightarrow \sigma^*_{xy}$ (35 %)	36633.9	34756.7	

Table S16. $CrN(trop)_2$ excited states for active space (9,9) and cc-pVDZ-DK basis set. Excitations from the ground state Kramers Doublet (KD) in cm^{-1} . Values computed with both CASSCF and NEVPT2 methods are reported and compared with experimental results (Fig. S26). The character and weight of the main component of each CAS wavefunction is reported in the second column.

	CAS(1,5)	NEV(1,5)	CAS(9,9)	NEV(9,9)	Exp.
g_{iso}	1.960	1.983	1.975	1.978	1.9875
$g_{//}$	1.900	1.955	1.924	1.934	1.9673
$g_{\perp}$	1.992	1.997	2.005	2.005	1.9976

Table S17. CrN(pyrdtc)$_2$ g-matrix values for DKH-def2-TZVPP basis set.
Computed values of g-matrix with both CASSCF and NEVPT2 methods and different active spaces. Results are compared with experimental results.

	CAS(1,5)	NEV(1,5)	CAS(9,9)	NEV(9,9)	Exp.
g_{iso}	1.960	1.982	1.974	1.979	1.9875
$g_{//}$	1.898	1.954	1.923	1.934	1.9673
$g_{\perp}$	1.992	1.997	2.005	2.005	1.9976

Table S18. CrN(pyrdtc)$_2$ g-matrix values for cc-pVDZ-DK basis set.
Computed values of g-matrix with both CASSCF and NEVPT2 methods and different active spaces. Results are compared with experimental results.

	CAS(1,5)	NEV(1,5)	CAS(9,9)	NEV(9,9)	Exp.
g_{iso}	1.960	1.982	1.974	1.978	1.9875
$g_{//}$	1.899	1.954	1.923	1.934	1.9673
$g_{\perp}$	1.992	1.997	2.005	2.005	1.9976

Table S19. CrN(pyrdtc)$_2$ g-matrix values for cc-pVTZ-DK basis set.
Computed values of g-matrix with both CASSCF and NEVPT2 methods and different active spaces. Results are compared with experimental results.

	CAS(1,5)	NEV(1,5)	CAS(9,9)	NEV(9,9)	Exp.
g_{iso}	1.959	1.982	1.974	1.978	1.9875
$g_{//}$	1.898	1.954	1.922	1.933	1.9673
$g_{\perp}$	1.992	1.997	2.005	2.005	1.9976

Table S20. CrN(pyrdtc)$_2$ g-matrix values for cc-pVQZ-DK basis set.
Computed values of g-matrix with both CASSCF and NEVPT2 methods and different active spaces. Results are compared with experimental results.

	CAS(1,5)	NEV(1,5)	CAS(9,9)	NEV(9,9)	Exp.
g_{iso}	1.958	1.971	1.967	1.971	1.9678
$g_{//}$	1.895	1.925	1.908	1.918	1.9415
$g_{\perp}$	1.990	1.995	2.001	2.001	1.9958

Table S21. CrN(trop)$_2$ g-matrix values for DKH-def2-TZVPP basis set.
Computed values of g-matrix with both CASSCF and NEVPT2 methods and different active spaces. Results are compared with experimental results.

	CAS(1,5)	NEV(1,5)	CAS(9,9)	NEV(9,9)	Exp.
g_{iso}	1.957	1.971	1.967	1.970	1.9678
$g_{//}$	1.894	1.924	1.907	1.918	1.9415
$g_{\perp}$	1.990	1.995	2.001	2.001	1.9958

Table S22. $CrN(trop)_2$ g-matrix values for cc-pVDZ-DK basis set.
Computed values of g-matrix with both CASSCF and NEVPT2 methods and different active spaces. Results are compared with experimental results.
